# Combined exposure to shrimp tropomyosin and microbial components triggers enhanced allergic and inflammatory responses *in vitro*

**DOI:** 10.3389/falgy.2025.1654600

**Published:** 2025-11-24

**Authors:** Fikirte Debebe Zegeye, Steen Mollerup, Mayes Alswady-Hoff, Annette Kuehn, Sabina Burla, Anne Straumfors, Johanna Samulin Erdem

**Affiliations:** 1 Department of Occupational Toxicology, National Institute of Occupational Health, Oslo, Norway; 2 Faculty of Medicine, University of Oslo, Oslo, Norway; 3 Department of Infection and Immunity, Luxembourg Institute of Health, Esch-sur-Alzette, Luxembourg; 4 INVITROLIZE, Esch-sur-Alzette, Luxembourg; 5 Faculty of Food Science and Technology, University of Agricultural Sciences and Veterinary Medicine of Cluj-Napoca, Cluj-Napoca, Romania

**Keywords:** allergy, additive effects, bioaerosol exposure, inflammation, occupational exposure, allergens, tropomyosin, microbial components

## Abstract

**Introduction:**

Workers in the shellfish industry face increased risks of allergy and asthma due to complex bioaerosol exposures in the workplace. This study aimed to assess whether combined exposure to the main components of these aerosols, specifically allergens and microbial agents, can potentiate inflammatory and allergic responses.

**Methods:**

THP-1 monocytes and advanced human alveolar co-cultures model ALIsens® were exposed to shrimp tropomyosin (0.0049, 1.3 and 2.6 µg/mL), and components from Gram-positive bacteria; lipoteichoic acid (0.25–4 µg/mL), and fungi; zymosan (6.25–100 µg/mL), either alone or in combination. The effects on the gene expression and protein secretion of chemokines and cytokines were assessed by RT-qPCR and ELISA or Luminex.

**Results:**

Combined exposure to tropomyosin and lipoteichoic acid resulted in increased *CCL20*, *CCL2, TNF* and *IL8* expression and CCL20 and TNF protein secretion in THP-1 cells, when compared to individual exposure. Similarly, tropomyosin combined with zymosan elicited a response pattern, characterised by increased expression and secretion of chemokines and cytokines in most of the tested combinations. Furthermore, the increased secretion of CCL20 and expression of *CCL2* following combined exposure to tropomyosin and lipoteichoic acid were confirmed in the alveolar co-culture model, while no effects in combination with zymosan were observed.

**Conclusion:**

These findings suggest that microbial components in shellfish industry bioaerosols may enhance the immunological responses caused by inhaled allergens in an additive manner, highlighting the need to minimise microbial contamination in workplaces where allergen exposure is prevalent.

## 1 Introduction

Global seafood production has grown steadily at an annual rate of 3.2%, reaching a record of over 185 million tonnes in 2022, and this sector now engages more than 61 million people worldwide (1). This growth is fuelled by the increasing awareness of the health benefits of consuming seafood (2, 3). Many seafood products require processing before consumption, typically involving butchering, washing, thawing, cooking, boiling, beheading, peeling, and grinding (4). These processing steps can aerosolise biological components into the air, creating complex mixtures of allergens, microorganisms, proteases, endotoxins and preservatives as a form of steam, vapour and dust (4, 5). As a result, there is growing concern over the health of processing workers, particularly regarding respiratory illnesses such as asthma and inhalant allergies. Indeed, occupational asthma is prevalent among seafood industry workers, reaching up to 36% in the shellfish industry (6–8). Clinical symptoms among shellfish-processing workers include a stuffy/runny nose and impaired lung function (9, 10). Importantly, shellfish-processing workers are exposed to a complex mix of bioaerosols containing allergenic proteins, microorganisms, endotoxins and other microbial ligands (9–12).

Among the allergenic proteins, tropomyosin is the major allergen in shrimp (13–17). Characterisation of workplace bioaerosols in shrimp processing plants revealed high levels of tropomyosin, while microbiome analysis of the same work environment identified Gram-positive bacteria and yeast-like fungi as the predominant microbial species (10, 12). Inhalation of these complex bioaerosols has been linked to respiratory health effects, including allergic reactions (18–20).

Upon inhalation, airborne allergens and microbial components interact with the respiratory epithelium, triggering a cascade of early immune events in the lungs. These interactions are initially detected by epithelial cells and resident macrophages, which coordinate the recruitment and activation of immune cells, including dendritic cells, eosinophils, and monocytes (21). Central to this process are T-helper type 2 (Th2) cells associated cytokines such as IL4, IL5, and IL13, which drive allergic inflammation and immunoglobulin E (IgE) production (21, 22). While exposure to allergens causes classical Th2 responses, the role of non-allergenic substances, such as microbial components, and their combined effects with allergens on respiratory health remains poorly understood. This is particularly relevant in occupational settings, such as shrimp processing, where workers are exposed to complex mixtures of allergenic proteins and microbial agents. Thus, the present study aimed to investigate the effects of combined exposure to the major shrimp allergen tropomyosin and representative microbial components from bacteria and fungi, focusing on key chemokines and cytokines involved in allergic and inflammatory responses including c-c motif chemokine ligand 20 (CCL20) and CCL2 (recruitment of monocytes and dendritic cells), as well as tumor necrosis factor (TNF) and interleukin 8 (IL8) (markers of acute inflammation). These biomarkers were selected to provide insight into early immune activation and pathways potentially relevant to allergic sensitisation in occupational bioaerosol exposure.

## 2 Materials and methods

### 2.1 Cell models and exposure

Human cell lines, monocytes THP-1, alveolar type II epithelial cells (A549), and endothelial cells (EA.hy926) were obtained from the American Type Culture Collection (Rockville, MD, USA). All cell lines were authenticated via DNA fingerprinting in 2024, and cells in culture were regularly screened for *mycoplasma* contamination.

**Figure 1:**
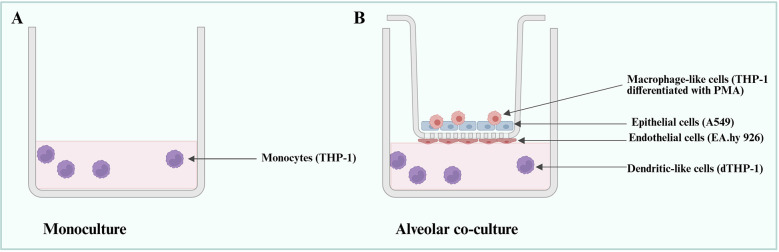
Simplified illustration of the cell models used in the study. **(A)** THP-1 cells in monoculture. **(B)** Alveolar co-culture model ALIsens® comprised of macrophages [phorbol 12-myristate 134-acetate (PMA)-differentiated THP-1], A549 cells, EA.hy926 cells and dendritic-like THP-1 (dTHP-1) cells. Created in https://BioRender.com.

Two cell model types were used, a monoculture of THP-1 cells and an alveolar co-culture, as illustrated in Figure 1.

For submerged exposure, THP-1 cells were seeded at a density of 1 × 10^6^ cells/mL in 6-well culture plates (Greiner Bio-One, Kremsmünster, Austria) and allowed to rest overnight. Following this resting period, the resuspended exposure materials were added directly to the cells. After a 24 h exposure, both the supernatant and cells were collected for downstream analysis.

The alveolar co-culture model ALIsens®, comprised of A549 cells, EA.hy926 cells, macrophage-like cells [phorbol 12-myristate 134-acetate (PMA)-differentiated THP-1], and non-differentiated THP-1 cells, hereafter referred to as dendritic-like THP-1 (dTHP-1) cells, was maintained under air-liquid interface (ALI) conditions as previously published (23). The ALI exposure mimics physiological breathing conditions by allowing the apical surface of airway epithelial cells to be directly exposed to airborne substances, enabling a more realistic assessment of cellular responses to inhaled particles, allergens, or microbial agents. All cells were maintained in their appropriate cell culture media at 37 °C and 5% CO_2_ (Supplementary Table S1).

In short, on day 0, THP-1 cells were plated at a density of 4 × 10^5^ cells/mL and were differentiated to macrophage-like cells using 2 ng/mL PMA (Sigma Aldrich Corporation, Missouri, USA) for 48 h. PMA medium was replaced on day 2 with fresh medium, and the incubation continued for 4 days. On the same day, EA.hy926 cells were seeded at a density of 2.4 × 10^4^ cells/cm^2^ on an inverted Transwell™ insert (4.5 cm² surface area; 5 μm pores; high-density PET membranes for 6-well plates, cellQART, Northeim, Germany). After 4 h, the inserts were inverted, and A549 (6 × 10^4^ cells/cm²) were seeded on the apical side. The plate was incubated for 3 days. On day 5, the medium was changed to a co-culture medium containing 10% FBS. PMA-differentiated THP-1 cells (2.4 × 10^4^ cells/cm²) and dTHP-1 (1 × 10^6^ cells/mL) were resuspended with a co-culture medium containing 1% FBS separately and were added to the apical side of the insert and the bottom of the wells, respectively. After 4 h of incubation, the medium from the apical compartment was removed, and the co-culture was maintained at ALI conditions overnight. The following day, the co-culture was exposed at ALI using the Vitrocell® Cloud Alpha MAX 6 well system (Vitrocell®, Waldkirch, Germany), and the cells were collected 24 h post-exposure for downstream analysis.

### 2.2 Model exposure agents and selection of doses

Shrimp tropomyosin protein (Pen m1) was expressed in *E coli* M15 and purified by column chromatography as reported earlier (24). The activity of the protein has been previously assessed by *in vitro* and *ex vivo* experiments (24, 25). For the remainder of the text, Pen m1 will be referred to as TM. Microbial components, lipoteichoic acid (LTA) purified from a Gram-positive bacterium, *Staphylococcus aureus* and zymosan (ZYM) from yeast-like fungi, *Saccharomyces cerevisiae,* were purchased from InvivoGen (San Diego, USA). All exposure agents were diluted in sterile water. For ALI exposure, the stock solutions were further diluted with 50% (v/v) sterile water in PBS (1X).

Concentrations of TM used for *in vitro* exposure were derived from measured workplace levels and typical employment durations in Norwegian shrimp processing plants. Specifically, the median (0.3 µg/m^3^) and maximum (7.07 µg/m^3^) airborne tropomyosin measured in two Norwegian shrimp processing plants and the respective average (3.3 years) and maximum (38 years) years of employment (emp.) previously reported for these workers (10) were used to represent the median and the worst-case scenarios. Assuming a total breathing volume of 3 m^3^ over an 8 h shift and considering an adult lung surface area of 140 m^2^, the corresponding TM concentrations were selected by adopting and modifying the lung deposition formula described by (51), This approach reflects a cumulative exposure effect integrating both exposure intensity and duration to approximate the total inhaled and deposited burden of tropomyosin that may occur over a working life time. While these parameters represent simplified assumptions that may not fully capture individual physiological variability or workplace conditions, they provide a standardised, conservative basis for estimating biologically and occupationally relevant *in vitro* concentrations.

$$\begin{array}{rl} & \;\mathrm{“Daily}\;\mathrm{Exposure}\;\mathrm{Dose”}\;\left(\mu \frac{g}{{\mathrm{cm}}^{2}}\right)\\ & \;=\mathrm{TM}\;\mathrm{Conc}.\left(\mu \frac{g}{{\mathrm{cm}}^{3}}\right)\times \mathrm{Breathing}\;\mathrm{volume}(3{\;m}^{3})/\mathrm{Lung}\;\mathrm{surface}\;\mathrm{area}\;(140{\;m}^{2}) \end{array}$$

$$\begin{array}{rl} & \mathrm{“Exposure}\;\mathrm{dose}\;\mathrm{over}\;\mathrm{emp}.\;\mathrm{perio}d”\left(\mu \frac{g}{{\mathrm{cm}}^{2}}\right)\\ & =\mathrm{Daily}\;\mathrm{Exposure}\;\mathrm{Dose}\;\;\left(\mu \frac{g}{{\mathrm{cm}}^{2}}\right)\;\times 5\;\left(\frac{days}{week}\right)\times 48\frac{weeks}{year}\times emp.\;(yrs) \end{array}$$

The selected concentrations of TM were (0.0049, 1.3 and 2.6 µg/mL) for THP-1 cells and equivalent concentrations of the first two concentrations for the co-culture alveolar model (0.00051 and 0.138 µg/cm^2^). Detailed TM dose calculations are provided in Supplementary Table S2. In addition, five concentrations of LTA (0.25, 0.5, 1, 2 and 4 µg/mL) for THP-1 cells and one dose for the co-culture (0.53 µg/cm^2^, corresponding to approximately 4 µg/mL), as well as five doses of ZYM (6.25, 12.5, 25, 50 and 100 µg/mL) and one dose for the co-culture (11.1 µg/cm^2^, corresponding to approximately 100 µg/mL) were selected based on previous *in vitro* studies (26, 27). Concentration details are provided in Table 1. Cells were exposed to TM alone or in combination with LTA or ZYM. All exposures were performed at subtoxic concentrations (Supplementary Figures S1,S2,S11), as assessed by the alamarBlue™ cell viability kit, according to the manufacturer's recommendations (ThermoFisher Scientific, Waltham, MA, USA). Concentrations resulting in viability levels below 85% were considered cytotoxic and not included in further analysis.

**Table 1:** Exposure concentrations used in the study.

Cell model types	Exposure agents	Exposure concentration used
Monoculture	TM	0.0049, 1.3 and 2.6 µg/mL
	LTA	0.25, 0.5, 1, 2 and 4 µg/mL
	ZYM	6.25, 12.5, 25, 50 and 100 µg/mL
Co-culture	TM	0.00051 and 0.138 µg/cm^2^
	LTA	0.53 µg/cm^2^
	ZYM	11.1 µg/cm^2^

### 2.3 Analysis of secreted proteins

The secretion of pro-inflammatory markers CCL20 and TNF was measured in the cell culture media 24 h post-exposure. Quantification was performed using human CCL20/MIP-3*α* and human TNF*α* enzyme-linked immunosorbent assay (ELISA) kits (R&D Systems, Minneapolis, MN, USA) according to the manufacturer's protocol. All samples were run in parallel, and absorbance was read at 450 nm using BioTek Synergy Neo2 Hybrid Multimode Reader (Agilent Technologies).

Cytokines and chemokines released from the alveolar co-culture model were measured using a custom multiplex human Luminex discovery assay (R&D Systems). The multiplex assay included CCL2, CCL20, colony-stimulating factor 2 (CSF2), surfactant protein D (SFTPD), and chitinase 3-like 1 (CHI3L1), with a sensitivity ranging from 3.3 to 38.1 pg/mL for the different cytokines and chemokines. Samples were analysed in parallel using the xMAP INTELLIFLEX instrument (R&D Systems).

### 2.4 Gene expression

Total RNA was isolated from both the THP-1 cells and the alveolar co-culture model using the total RNA purification kit (Norgen Biotek Corp., Ontario, Canada), according to the manufacturer's instructions. For the alveolar co-culture model, RNA was collectively extracted from all the cell types present on the culture insert, comprising A549, EA. hy926 and PMA-differentiated THP-1 cells. In addition, RNA was extracted separately from the dTHP-1 cells present in the basolateral compartment. Concentrations and purity of RNA were quantified using a Nanodrop 2000 spectrophotometer (ThermoFisher Scientific) and Qubit 4 Fluorometer Invitrogen (ThermoFisher Scientific).

RNA isolated from THP-1 cells was reverse transcribed using a qScript cDNA synthesis kit (Quanta BioSciences, Beverly, MA, USA), following the manufacturer's instructions. Expression analysis of *CCL20, CCL2,* interleukin 1 receptor-like 1 *(IL1RL1), CHI3L1, TNF* and *IL8* was performed by SYBR Green technology on the QuantStudio 5 Real-Time PCR System (Applied Biosystems, ThermoFisher Scientific), using predesigned KiCqStart™ Primers (Sigma Aldrich). The primer ID and sequences are provided in Supplementary Table S3.

Gene expression analysis on co-culture samples was performed using RT2 First Strand cDNA Kit and a custom asthma and allergy RT2 Profiler PCR Array (Qiagen, Hilden, Germany) on the QuantStudio 5 Real-Time PCR System (ThermoFisher Scientific). The custom array contained 24 genes: *ADRB2, CCL11, CCL17, CCL2, CCL20, CCL26, CHI3L1, CRLF2, CSF1, CSF2, IL13, IL1RL1, IL25, IL33, IL4, IL5, MRC1, MS4A2, POSTN, PTGDR2, STAT6, TNFRSF4, TNFSF4* and *TSLP.* Full gene names can be found in Supplementary Table S4.

The gene expression was calculated by the ΔΔCt method. For normalisation, five housekeeping genes: beta-2-microglobulin (*B2M*), hypoxanthine phosphoribosyl transferase 1 (*HPRT1*)*,* TATA-box binding protein (*TBP*)*,* hydroxymethylbilane synthase (*HMBS*)*,* and transferrin receptor (*TFRC*), were included in the analyses. The gene expression data were normalised to the geometric mean of the most stable housekeeping genes, which were *B2M* and *HPRT1* for the monocultures, and *TBP, HMBS, HPRT1,* and *TFRC* for the co-culture samples. All housekeeping genes selected for normalisation had intra-assay variability below 0.2 CT. Further comparisons between individual and combined exposure were performed on the fold change values against the vehicle control values.

### 2.5 Flow cytometry

Co-culture model performance and reproducibility were assessed using flow cytometry (Supplementary Figure S3), consistent with approaches previously reported in conference proceedings (28). Co-culture was treated with positive control, a mix of recombinant thymic stromal lymphopoietin protein (TSLP) (1.11 ng/cm^2^) and lipopolysaccharide (LPS) (550 ng/cm^2^) (Sigma Aldrich), or negative controls (incubator control and vehicle control). dTHP-1 cells were harvested from the basolateral compartment of the wells and then stained with fluorescence-labelled monoclonal antibodies, along with isotype-matched controls (mouse IgG1). The following antibodies were used: BB515 anti-CD54 (clone HA58) and APC anti-TSLPR (clone 1F11), purchased from BD Biosciences (Heidelberg, Germany). Surface marker expression was assessed using CytoFLEX (Beckman Coulter Life Sciences, Indianapolis, IN, USA) and analysed with CytExpert software. Cells that stained positive with fixable viability stain 510 (Heidelberg, Germany) (indicating dead cells) were excluded from the analysis. Results were shown as percentage positive cells as determined by gating against the unstained and isotype controls.

### 2.6 Statistical analysis

Statistical analysis was conducted using GraphPad Prism version 10 or R version 4.4.1. All data were log-transformed, and group comparisons were made using a one-way ANOVA followed by Sidak's multiple comparisons test. Bliss independence analysis was applied to evaluate additive and synergistic interactions based on the assumption of independent mechanisms of action. Deviations of the observed combination effect from the expected additive effect were quantified. While percentage differences greater than 30% were noted as potentially indicative of synergy, only statistically significant deviations (*p* < 0.05) were interpreted as evidence of true synergy. Data transformation and the type of analysis used are specified in the figure legends of each result. Raw data and *p*-values from all the analyses are available in Supplementary Data 2.

## 3 Results

### 3.1 Dose-dependent effects of LTA, ZYM and TM on allergic and inflammatory biomarkers

**Figure 2:**
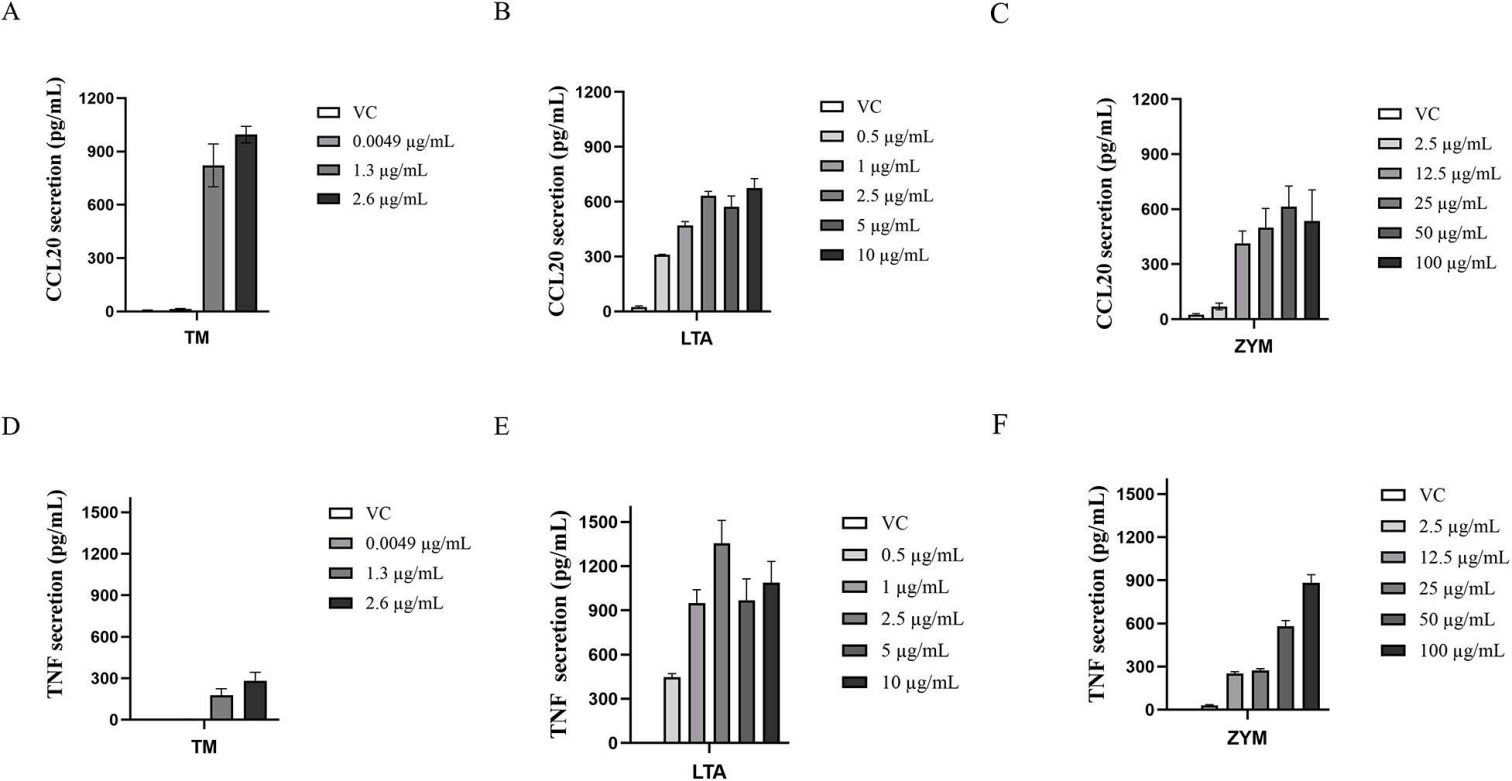
Release of inflammatory markers. THP-1 cells were exposed to three different concentrations of shrimp tropomyosin (TM) and five concentrations of lipoteichoic acid (LTA), zymosan (ZYM) for 24 h and assessed for release of CCL20 **(A–C)** and TNF **(D–F)**. The bars represent the mean ± SEM, *n* = 2–5. VC indicates vehicle control.

Individual exposures to TM, LTA, and ZYM resulted in a dose-dependent increase in the secretion of CCL20 and TNF (Figure 2). Based on this result, concentrations of LTA and ZYM were selected for further analysis.

Individual exposure of THP-1 cells to the selected LTA concentrations (0.25, 0.5, 1.0, 2.0, and 4.0 µg/mL) resulted in a dose-dependent increase in the expression of *CCL20*, *CCL2*, *TNF* and *IL8* that reached up to a 341-fold increase relative to the vehicle control (Supplementary Data 2). Similarly, individual exposure to the five ZYM concentrations (6.25, 12.5, 25, 50, and 100 µg/mL) resulted in a dose-dependent increase in the first four ZYM concentrations for *CCL20* and *CCL2* (Supplementary Data 2). Other markers showed low expression or inconsistent data (Supplementary Data 2).

In the alveolar co-culture model, individual exposure to LTA (0.53 µg/cm^2^) resulted in an 18 and 15-fold increase in *CCL20* and *CCL2,* respectively, relative to the vehicle control (Supplementary Data 2). ZYM exposure also resulted in a >2-fold increase in these markers relative to the vehicle control (Supplementary Data 2).

### 3.2 LTA in combination with TM increases allergic and inflammatory signalling in THP-1 cells

Since combined exposure to LTA with the lowest TM concentration (0.0049 µg/mL) did not increase CCL20 or TNF secretion, nor significantly affect most other markers’ expression, the effects observed at higher TM concentrations (1.3 and 2.6 µg/mL) with LTA will be discussed in more detail.

**Figure 3:**
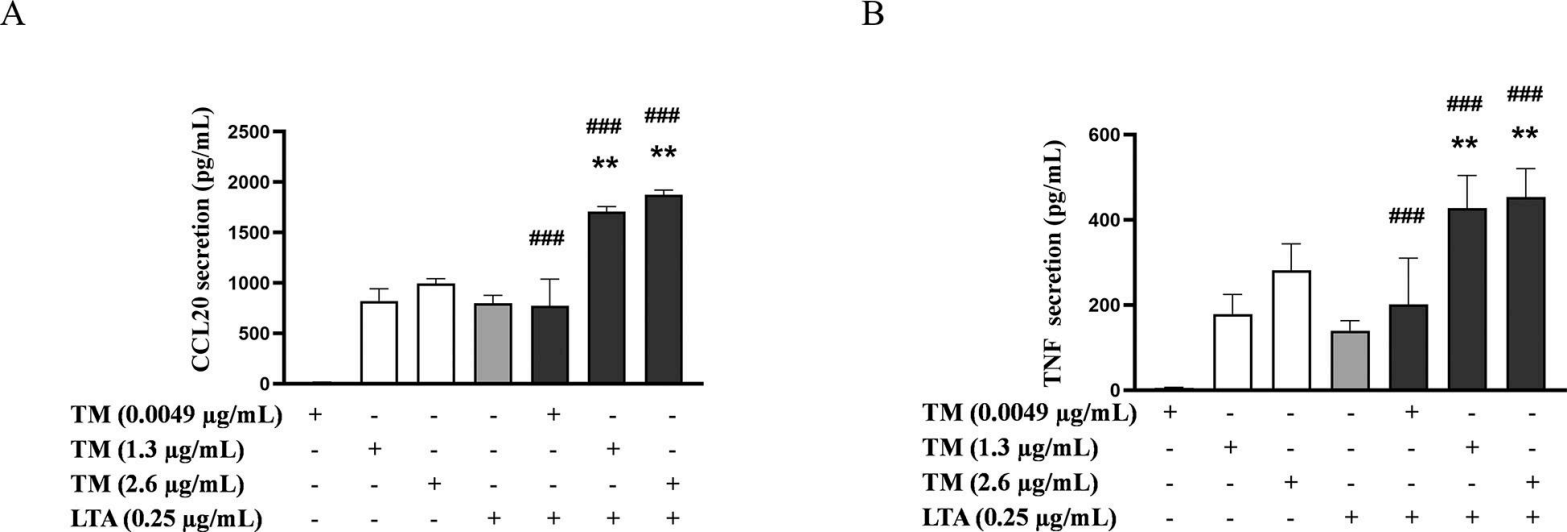
Effects of combined exposure on the secretion of proinflammatory markers in THP-1 cells following 24 h of exposure to shrimp tropomyosin (TM) and lipoteichoic acid (LTA). **(A)** CCL20 and **(B)** TNF. The bars represent the mean ± SEM, *n* = 3. One-way ANOVA with Sidak's multiple comparison test was performed on log-transformed data. Significant differences between LTA and combined exposure are indicated by *, TM and combined exposure indicated by #, ** or ## *p* < 0.005 and ### *p* < 0.001.

Combined exposure of LTA and TM (1.3 and 2.6 µg/mL) resulted in an increased secretion of CCL20 and TNF already at the lowest LTA concentration tested (0.25 µg/mL) (Figures 3A,B). This effect was consistent at the higher concentrations tested (Supplementary Figures S4A,B). For TNF, the combined exposure resulted in a significant additive effect at almost all combinations, except for TM (2.6 µg/mL) and LTA (4 µg/mL), whereas for CCL20, an additive effect was observed only at LTA concentrations of 0.25 and 4 µg/mL (Supplementary Figures S4A,B).

**Figure 4:**
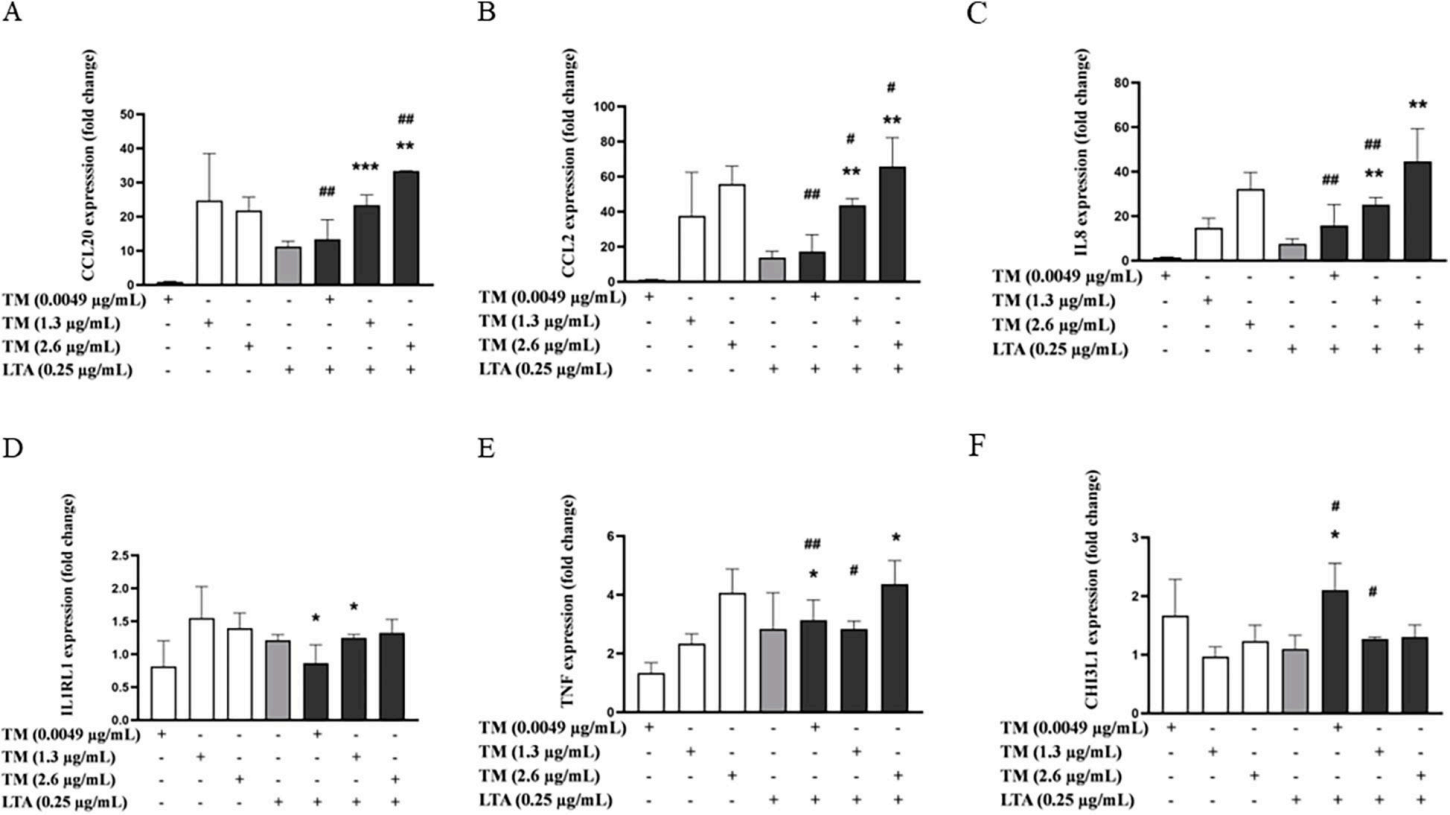
Effects of combined exposure on the expressions of proinflammatory markers in THP-1 cells following 24 h of exposure to shrimp tropomyosin (TM) and lipoteichoic acid (LTA). **(A)** *CCL20*, **(B)** *CCL2*, **(C)** *IL8*, **(D)** *IL1RL1*, **(E)** *TNF* and **(F)** *CHI3L1*. The bars represent the mean ± SEM, *n* = 3. One-way ANOVA with Sidak's multiple comparison test was performed on log-transformed data. Significant differences between LTA and combined exposure are indicated by *, TM and combined exposure indicated by #, * or # *p* < 0.05, ** or ## *p* < 0.005 and *** *p* < 0.001.

The effects of the combined exposure of the lowest LTA concentration with TM (1.3 and 2.6 µg/mL) on chemokine and cytokine expression were less consistent (Figure 4). Thus, while trends towards increased expression were observed mainly for *CCL20*, *CCL2* and *IL8* already at the lowest LTA concentration, only *CCL2* was consistently upregulated at both TM concentrations (Figures 4A–C). For *CCL2*, combined exposure resulted in the highest induction in expression at 1.0 µg/mL LTA, where it exceeded the additive effect by over 30% suggesting possible synergistic interactions (Supplementary Figure S5A). For *CCL20* and *IL8,* a trend of increased expression following combined exposure was observed for most concentrations tested, with additive effects noted for *CCL20* and *IL8* in cells exposed to LTA in combination with 2.6 µg/mL TM (Supplementary Figures S5B–S6A). For *IL1RL1,* enhanced expression was observed only at LTA concentrations >2.0 µg/mL (Supplementary Figure S6B). The effect of combined exposure on *TNF* and *CHI3L1* expression was complex, as low concentrations of both LTA and TM, in combination, generally resulted in increased expression of the markers. However, both LTA and TM at high concentrations surprisingly led to a reduction in the expression of these genes (Supplementary Figure S7).

### 3.3 ZYM in combination with TM enhances allergic and inflammatory signalling in THP-1 cells

**Figure 5:**
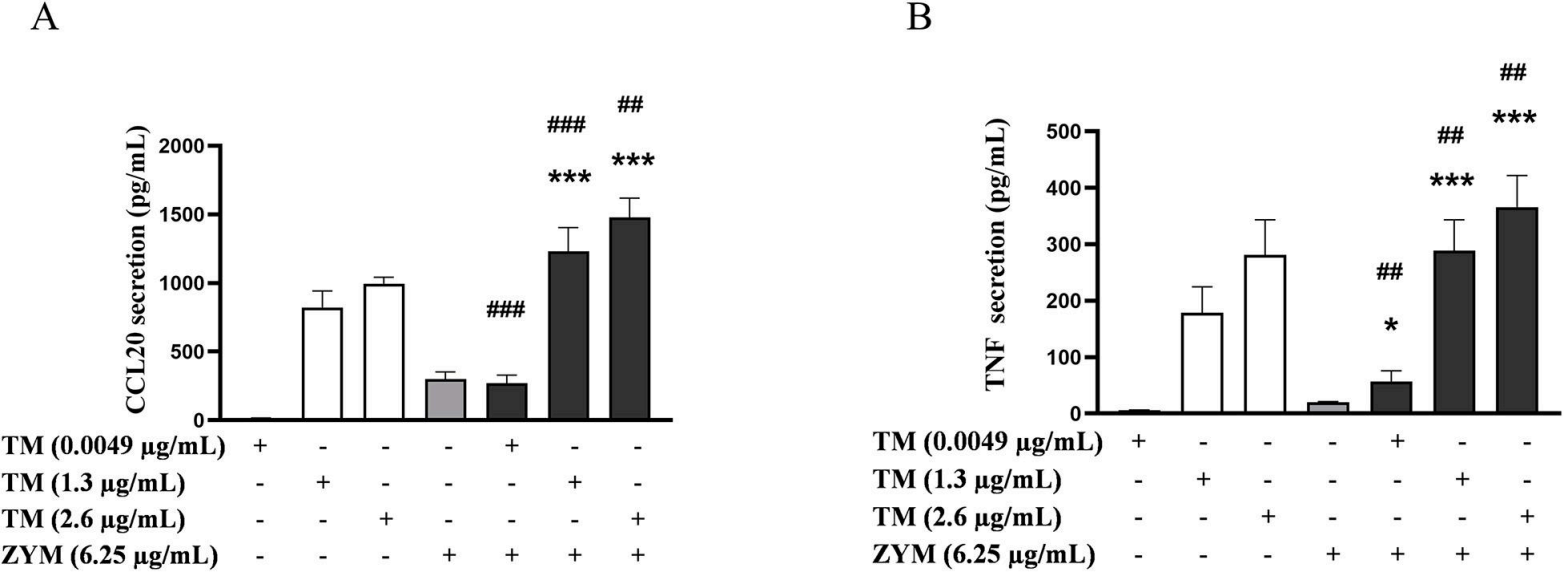
Effects of combined exposure on the secretion of proinflammatory markers in THP-1 cells following 24 h of exposure to shrimp tropomyosin (TM) and zymosan (ZYM). **(A)** CCL20 and **(B)** TNF. The bars represent the mean ± SEM, *n* = 3. One-way ANOVA with Sidak's multiple comparison test was performed on log-transformed data. Significant differences between ZYM and combined exposure are indicated by *, TM and combined exposure indicated by #, * *p* < 0.05, ## *p* < 0.005 and *** or ### *p* < 0.001.

The combined exposure of ZYM with TM resulted in enhanced secretion of both CCL20 and TNF compared to ZYM and TM alone, already at the lowest ZYM concentration tested (Figure 5). Increased TNF secretion was consistently observed for almost all combinations, while for CCL20, the effect was most prominent at the lower concentrations of ZYM (6.25–25 µg/mL), primarily in combination with TM (1.3 and 2.6 µg/mL) (Supplementary Figure S8).

**Figure 6:**
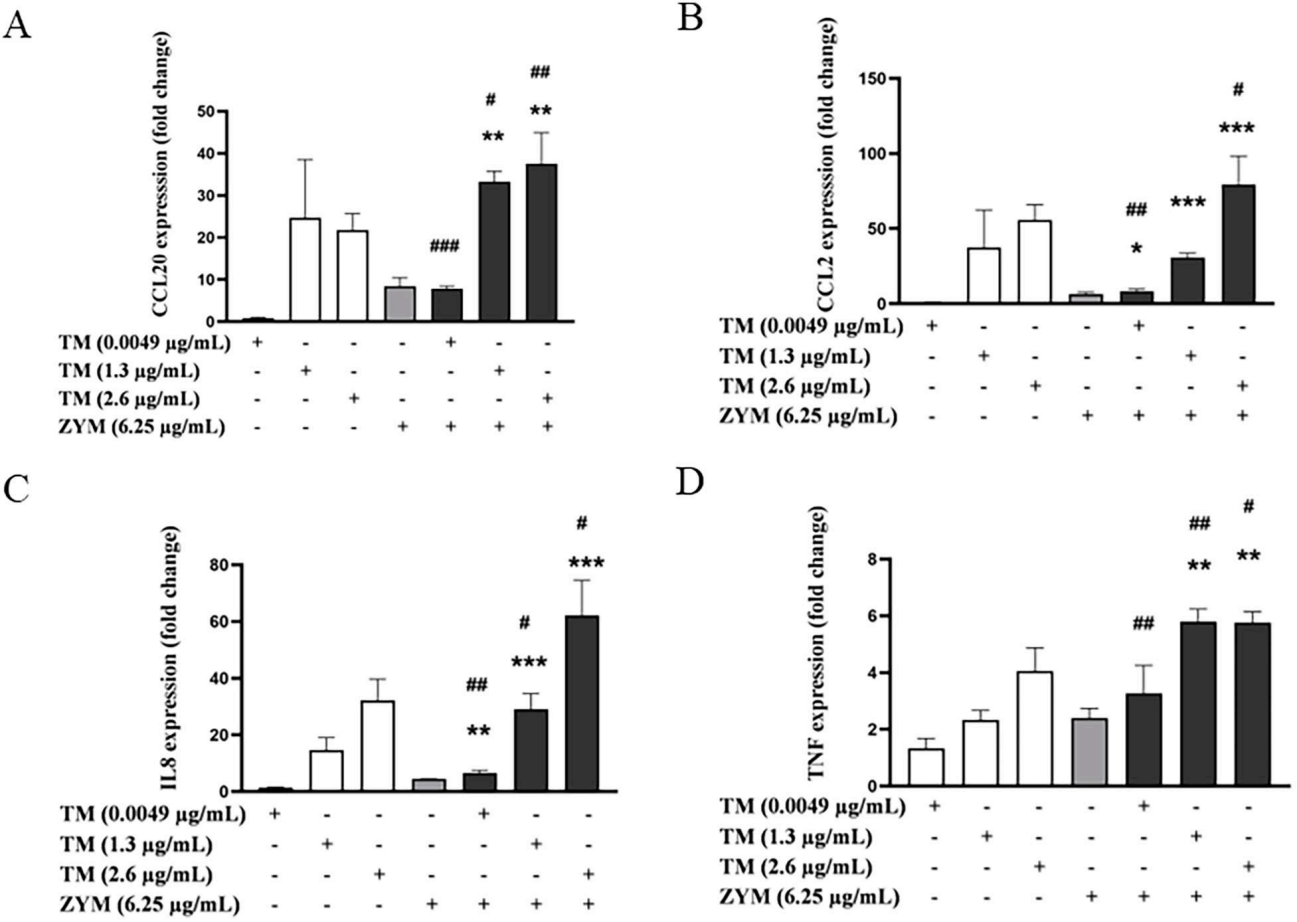
Effects of combined exposure on the expressions of proinflammatory markers in THP-1 cells following 24 h of exposure to shrimp tropomyosin (TM) and zymosan (ZYM). **(A)** *CCL20*, **(B)** *CCL2*, **(C)** *IL8* and **(D)** *TNF*. The bars represent the mean ± SEM, *n* = 3. One-way ANOVA with Sidak's multiple comparison test was performed on log-transformed data. Significant differences between ZYM and combined exposure are indicated by *, TM and combined exposure indicated by #, # *p* < 0.05, ** or ## *p* < 0.005 and *** *p* < 0.001.

The combined exposure of TM (1.3 and or 2.6 µg/mL) with ZYM resulted in increased expression of *CCL20*, *CCL2*, *IL8* and *TNF* genes, already at the lowest tested concentration (6.25 µg/mL) (Figure 6). Furthermore, the combination of TM (1.3 and 2.6 µg/mL) with ZYM resulted in a significant or near-significant increase in the expression of *CCL20* for all ZYM concentrations tested. Notably, the effects were large and additive (>30%) in combinations with the highest ZYM concentration (100 µg/mL), indicating possible synergistic effects (Supplementary Figure S9A). Similarly, an overall trend to increased *TNF* expression was also observed; however, this did not reach significance for most combinations (Supplementary Figure S9B). The combined exposure of the highest concentration of TM with ZYM also resulted in increased or additive effects on the *CCL2* expression for all ZYM concentrations (Supplementary Figure S10A). Whereas, for IL8, the expression was increased following combined exposure, especially at the lowest concentrations of ZYM (6.25 and 12.5 µg/mL) combined with all TM concentrations tested (Supplementary Figure S10B). Finally, only minor effects on *IL1RL1* and *CHI3L1* expression were observed, and since the overall changes in expression remained below a 2-fold increase compared to the vehicle control, these effects were not considered biologically significant (Supplementary Figure S11).

### 3.4 Assessment of combined effects in a human alveolar co-culture model

**Figure 7:**
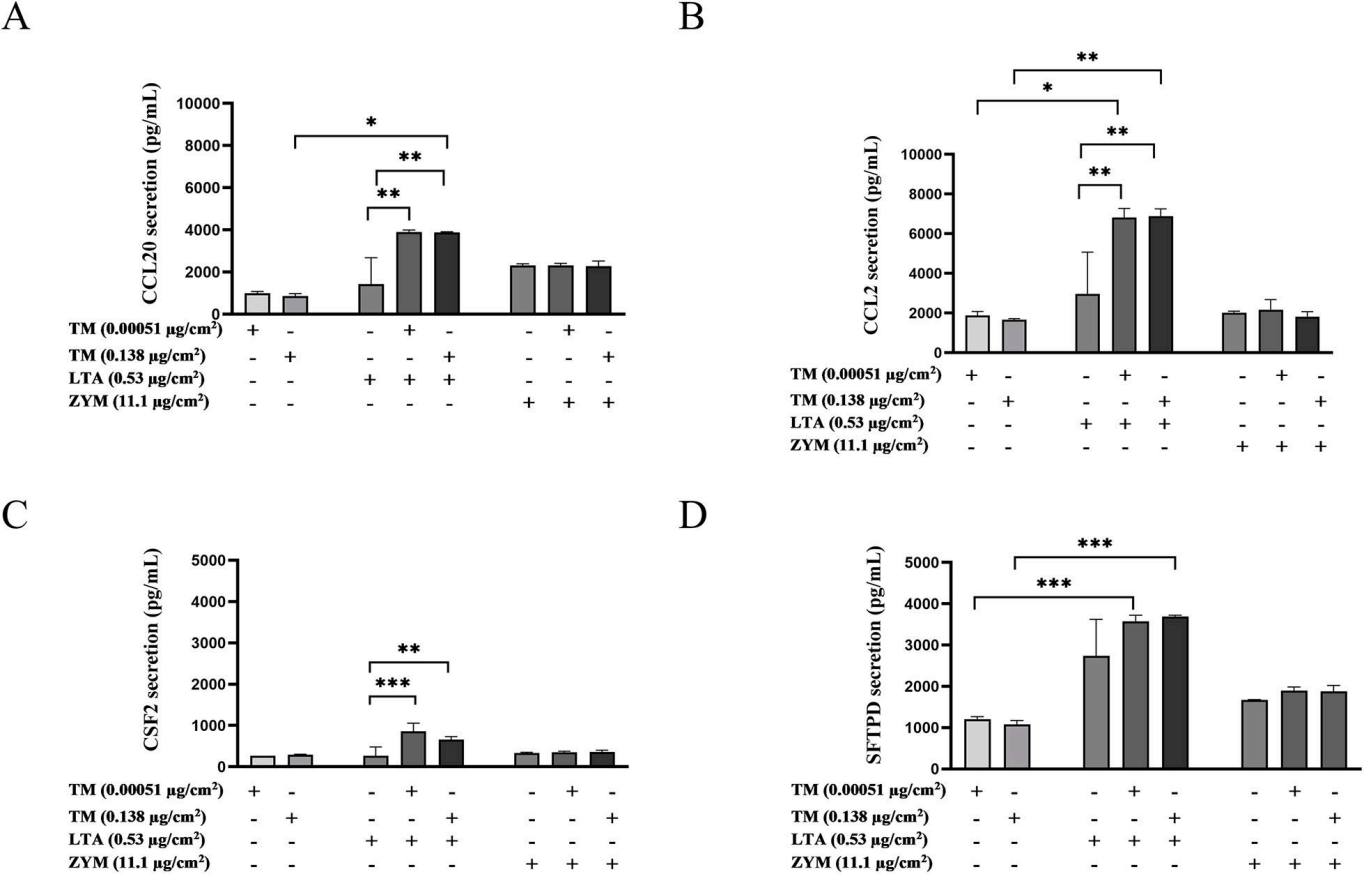
Secretion of markers related to inflammation, allergy and asthma from an alveolar co-culture model. **(A)** CCL20, **(B)** CCL2, **(C)** CSF2, and **(D)** SFTPD release were assessed after 24 h exposure to shrimp tropomyosin (TM), lipoteichoic acid (LTA), or zymosan (ZYM) alone and TM in combination with LTA or ZYM. The bars represent the mean ± SEM, *n* = 3. One-way ANOVA with Sidak's multiple comparison test was performed on log-transformed data. Significant differences compared to individual exposures are indicated by * *p* < 0.05, ** *p* < 0.001, and *** *p* < 0.0001.

The effects of selected subtoxic combinations of TM with LTA or ZYM (Supplementary Figure S12) were assessed in an alveolar co-culture model previously established to evaluate inflammatory and allergic responses. In this model, combined exposure to TM (0.138 µg/cm^2^) and LTA (0.53 µg/cm^2^) resulted in increased secretion of CCL20 compared to individual exposures (Figure 7A). Similarly, combined exposure of TM (0.0051 and 0.138 µg/cm^2^) with LTA (0.53 µg/cm^2^) resulted in enhanced CCL2 secretion compared to the individual exposure of LTA or TM (Figure 7B). The combined exposure of TM and LTA showed differential effects on CSF2 and SFTPD secretion, being significant compared to LTA alone (Figure 7C) and to TM alone (Figures 7D). Moreover, the combination of TM and ZYM did not result in any changes in the secretion of the proteins analysed. Similarly, no effects were observed for the combined exposures on the expression of other genes involved in inflammation, allergy, and asthma (Supplementary Figure S13).

## 4 Discussion

Occupational exposure to complex bioaerosols is associated with an increased risk of allergy and chronic pulmonary effects, including occupational asthma and rhinitis (7, 20, 29). However, the combined immunological effects of allergens and microbial components remain poorly understood. Workers in the shrimp processing industry are exposed to relatively low levels of protein, and endotoxin levels well below the occupational exposure limits (OEL) (30). However, chronic or repeated exposure to allergenic proteins and microbial components may increase chronic inflammatory responses and sensitisation (7, 29). Cumulative exposure metrics are well-established in occupational research and particularly relevant for bioaerosol exposures, reflecting the biologically effective dose associated with repeated inhalation of bioaerosols over years (31–33). This study demonstrated that combined exposure to shrimp TM at doses relevant for work-related chronic exposure and the microbial components LTA or ZYM resulted in increased secretion of chemokines and pro-inflammatory markers, specifically CCL20 and TNF. Furthermore, it enhanced the expression of biomarkers related to allergic and inflammatory processes, including *CCL20*, *CCL2*, *IL1RL1*, *TNF*, and *IL8*.

The increased secretion of CCL20 and enhanced expression of *CCL20* and *CCL2* chemokines in THP-1 cells following combined exposure suggest that the exposure may trigger an inflammatory response involving the recruitment of monocytes, dendritic cells and Th17 cells. While these chemokines are not specific markers of allergic Th2 cell-driven allergenic inflammation, they play a crucial role in both the development of airway inflammation and the late stages of allergic responses (21). Enhanced levels following combined exposure may have implications on the progression and severity of occupational airway diseases and asthma. Clinical studies have also shown that elevated IL8 levels are associated with more severe asthma phenotypes and contribute to systemic inflammation (34, 35). Thus, while these findings cannot be directly related to the present *in vitro* data, they highlight the potential importance of IL8 in the progression of airway disease. Furthermore, the observed pronounced upregulation of CCL20 expression and secretion may indicate the involvement of Th17-type responses, which are typical in host defence against bacteria and fungi and are relevant in occupational asthma linked to microbial exposure (36). Th17 responses are typically dominated by IL17, IL22, and CSF2 cytokines and are associated with CCL20 and TNF responses, which lead to recruitment of Th17 cells and amplification of inflammation (36, 37). As such, the enhanced TNF secretion and expression observed in THP-1 cells following combined exposure may further suggest potentiation of immune responses, which is essential in the development of chronic airway inflammation. Similarly, combined exposure to house dust mite (HDM) allergen Der p 2 and lipopolysaccharide (LPS) led to enhanced TNF and IL8 release from human whole blood cells, suggesting that contamination of allergens may be important in the progression of allergic inflammatory responses (38–40).

Moreover, Uzunoglu (41) reported that peptidoglycan, a cell wall component of Gram-positive bacteria, can trigger bioaerosol-induced inflammation via the secretion of TNF and CSF2, along with the recruitment of mast cells into the alveolar compartment (41). In line with this, it is becoming increasingly evident that the matrix of inhaled allergens is important in determining their sensitisation potential, and extrinsic compounds such as viruses and airborne contaminants may have immunomodulating effects (42). For pollen, LPS has been acknowledged as an important modulator of allergic sensitisation (43, 44). Similarly, Gram-positive bacteria have been shown to affect the maturation of dendritic cells derived from donors allergic to grass pollen (45).

Although combined exposure generally resulted in enhanced expression of most of the assessed markers, *TNF* and *CHI3L1* expression were surprisingly reduced at higher concentrations of the TM and LTA combination. Importantly, these changes were not accompanied by alterations in cell viability. The mechanisms underlying this observation remain unclear and require further investigation. A potential explanation is receptor or signalling desensitisation, whereby saturation or internalisation of receptors or downstream signalling components leads to diminished transcriptional activation (46, 47). Another possibility is the induction of endogenous negative regulators of inflammation, such as suppressor of cytokine signalling proteins or anti-inflammatory cytokines (e.g., IL10), which are known in many systems to feedback and suppress pro-inflammatory cytokine production (48). These possibilities highlight the complexity of immune responses to combined exposures and underscore the need for further studies to better understand the specific molecular pathways involved.

Finally, *IL1LR1*, which plays a central role in allergic inflammation and asthma as a key receptor for IL33, was upregulated following combined exposure to TM and microbial components. IL33 is an alarmin which is involved in the activation of immune cells important in Th2 responses and allergic inflammation, and the IL33/IL1LR1 axis is critical in susceptibility to asthma (49). Thus, the increased expression of *IL1RL1* observed following combined exposure to TM, especially the bacterial component LTA, further illustrates that the complexity of bioaerosols may affect pulmonary inflammatory responses and contribute to the exacerbation of respiratory symptoms.

While these results suggest that microbial bioaerosol contaminants may contribute to or modulate airway responses to allergens, the limitations of the THP-1 monoculture model should be taken into consideration when interpreting the findings. To address this, the human alveolar co-culture model ALIsens®, commonly used to assess the effects of sensitising and irritating chemicals on allergic sensitisation and inflammation, was additionally utilised (23). In this physiologically relevant *in vitro* model, the additive effects on the secretion of CCL20 and CCL2 were confirmed for combined exposure of TM and LTA. The increased secretion of these markers was observed at very low TM concentrations. While many other markers relevant for allergic responses and Th2 responses were included and assessed on both the alveolar co-culture model and THP-1 cells, no significant effects were observed following combined exposure, illustrating that CCL20 and CCL2 may be promising candidates for *in vitro* biomarkers to assess allergic and inflammatory responses to bioaerosols. Furthermore, elevated plasma levels of CCL20 have been demonstrated among Norwegian shrimp-processing workers, with a correlation between measured CCL20 plasma levels and levels of total protein exposure (10). Altogether, this provides evidence for the potential suitability of CCL20 as a biomarker of effect in occupational settings with allergen-containing exposures. Unfortunately, CCL2 was not analysed in the study by Zegeye et al., and thus the potential importance of CCL2 as a biomarker of effect for occupational exposure needs to be further validated.

Surprisingly, the effects of combined exposure with ZYM could not be confirmed in the alveolar co-culture model. While the ZYM and TM combination generally resulted in milder effects than LTA and TM co-exposure, this divergence is unlikely due to technical limitations, as both positive and negative controls performed as expected. Instead, it may reflect biological differences between the two models: cell interactions in the co-culture may attenuate responses that are otherwise amplified in THP-1 cells alone. At the same time, *in vitro* models do not allow for the potentiation of effects by the recruitment of additional inflammatory cells, as expected *in vivo*. Thus, while *in vitro* models cannot fully replicate the complexity of human responses or account for long-term exposure effects, a potential immunomodulating effect of fungal components on allergic responses to complex bioaerosols cannot be ruled out. Interestingly, enhanced inflammatory responses under combined exposure of fungal spores from *Avicularia versicolor* and HDM allergen have been shown in a murine model of asthma, which demonstrated a markedly increased asthma phenotype in exposed animals, characterised by increased airway hyperresponsiveness, eosinophilic and neutrophilic inflammation, and extensive lung tissue pathology, compared to individual exposures to either fungi or HDM alone (50). This implies that fungal contamination may be of importance in the assessment of health effects related to the inhalation of allergen-containing bioaerosols, further illustrating the need for more research in this field.

### 4.1 Conclusion

The findings of this study suggest that combined exposure to airborne allergens and microbial components may exacerbate airway inflammation and allergic responses even at low concentrations. The complexity of immune responses to multiple stimuli was illustrated specifically by CCL20 responses, which showed additive or even potentially synergistic effects following combined exposure.

Altogether, our findings suggest that individuals with simultaneous exposure to allergens and bacterial or fungal components may be at risk of experiencing more severe inflammatory responses, which could potentially exacerbate asthma symptoms. However, while cumulative dosing is commonly used in occupational research and the utilised doses were based on highly relevant median and worst-case occupational exposure scenarios among Norwegian shrimp processing workers, the models and exposure metrics used cannot fully capture the complexity and dynamics of chronic inflammatory responses. This highlights the need for additional research in the field and consideration of targeted interventions that account for the complexity of bioaerosols when managing allergic airway diseases in environments prone to allergens.

## Data Availability

The original contributions presented in the study are included in the article/Supplementary Material, further inquiries can be directed to the corresponding author/s.
