# Supplementary material for: Combined exposure to shrimp tropomyosin and microbial components triggers enhanced allergic and inflammatory responses in vitro
Source: Front Allergy. 2025 Nov 24;6:1654600. doi: 10.3389/falgy.2025.1654600 (PMC12683351; doi:10.3389/falgy.2025.1654600)
Supplement: Supplementary file 1 [file Datasheet1.pdf]

Supplementary data 1: Results

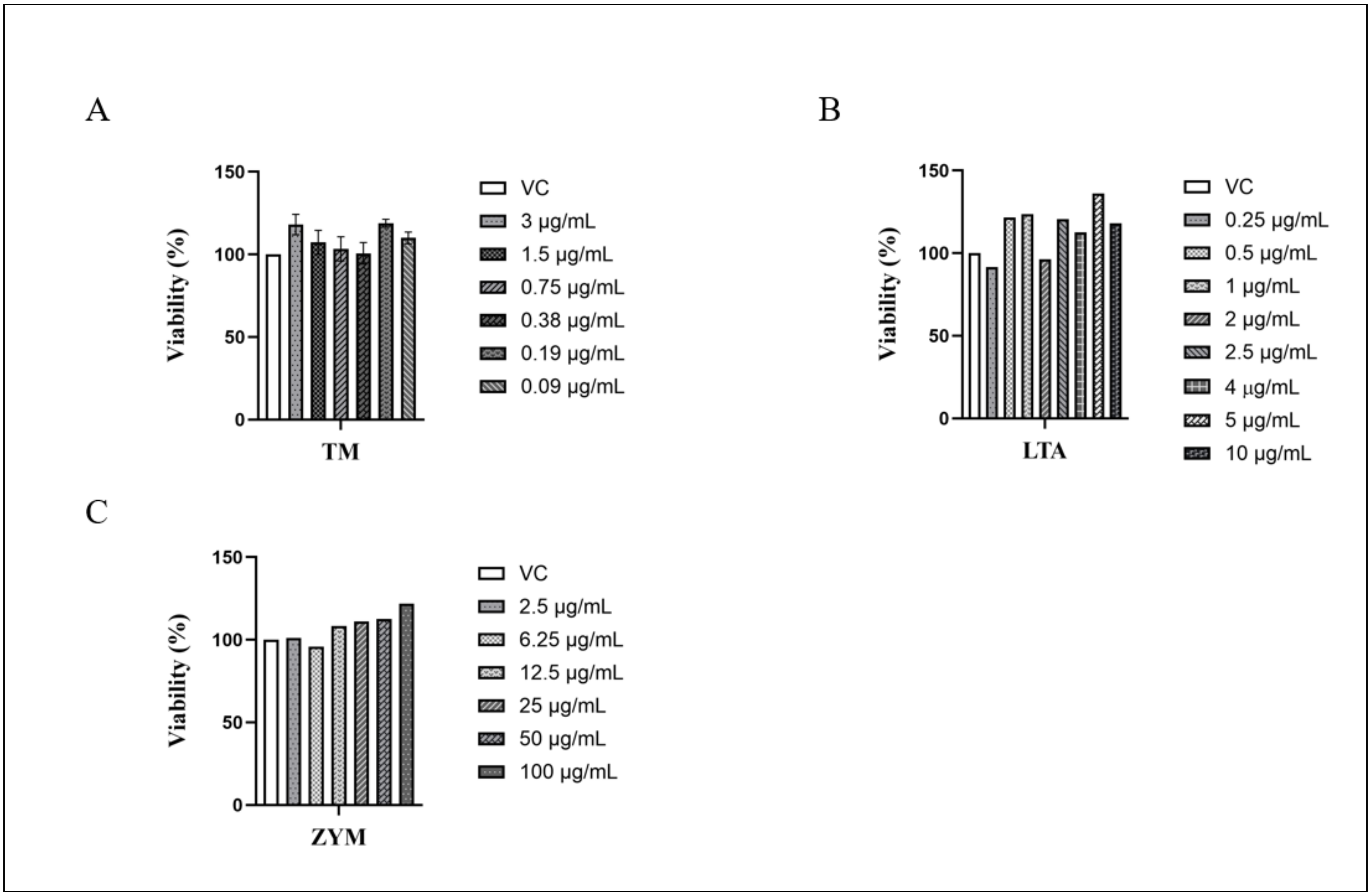

Figure S1 Viability of THP-1 cells after individual exposures. Cells were subjected to five-seven different concentrations of (A) Tropomyosin (TM), (B) lipoteichoic acid (LTA) and (C) zymosan (ZYM) for 24 h. The viability is presented as a percentage relative to the vehicle control (VC).

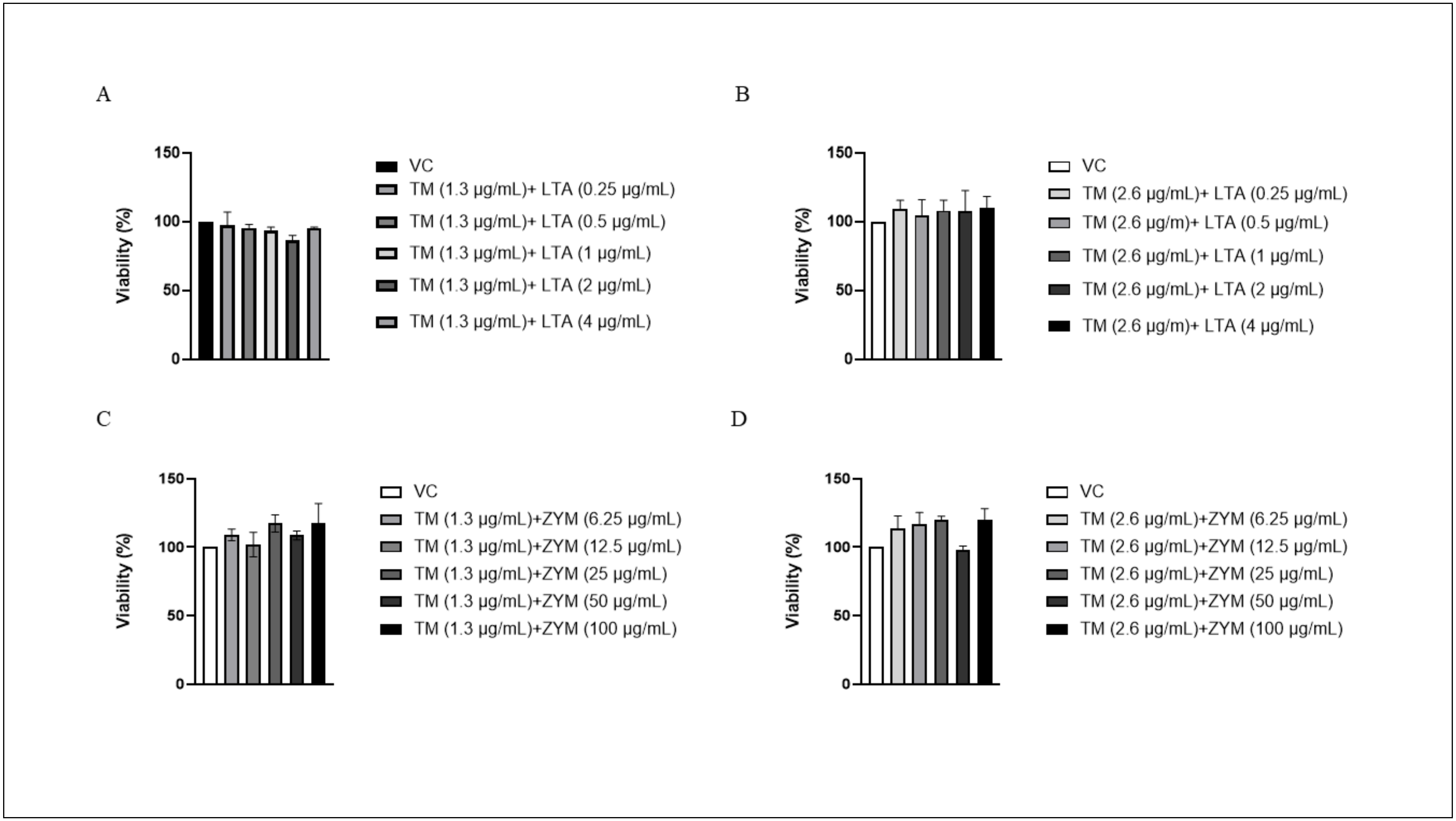

Figure S2 Viability of THP-1 cells after combined exposure to two different concentrations of shrimp tropomyosin (TM) and lipoteichoic acid (LTA) or Zymosan (ZYM) A) TM (1.3 µg/mL) with LTA (0.25, 0.5, 1, 2 and 4 µg/mL), B) TM (2.6 µg/mL) with LTA (0.25, 0.5, 1, 2 and 4 µg/mL), C) TM (1.3 µg/mL) combined with ZYM (6.25, 12.5, 25, 50 and 100 µg/mL) and D) TM (2.6 µg/mL) combined with ZYM (6.25, 12.5, 25, 50 and 100 µg/mL).VC - vehicle control.

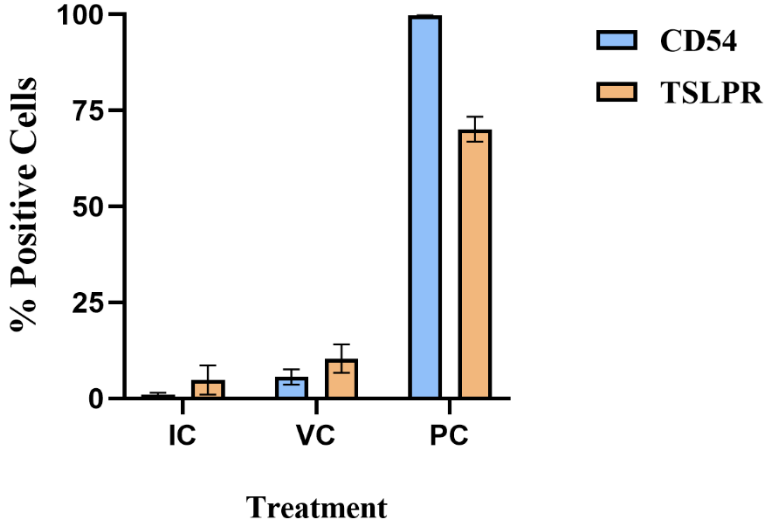

Figure S3 Co-culture model performance assessment by flow cytometry. Positive cells of dendritic-like THP-1 (dTHP-1) cells exposed to positive control, a mix of a recombinant protein thymic stromal lymphopoietin (TSLP) (1.11 ng/cm^2) and lipopolysaccharide (LPS) (550 ng/cm^2), and were assessed for surface marker expression of CD54 and thymic stromal lymphopoietin receptor (TSLPR), IC: incubator control, VC: vehicle control, PC: positive control, n=3.

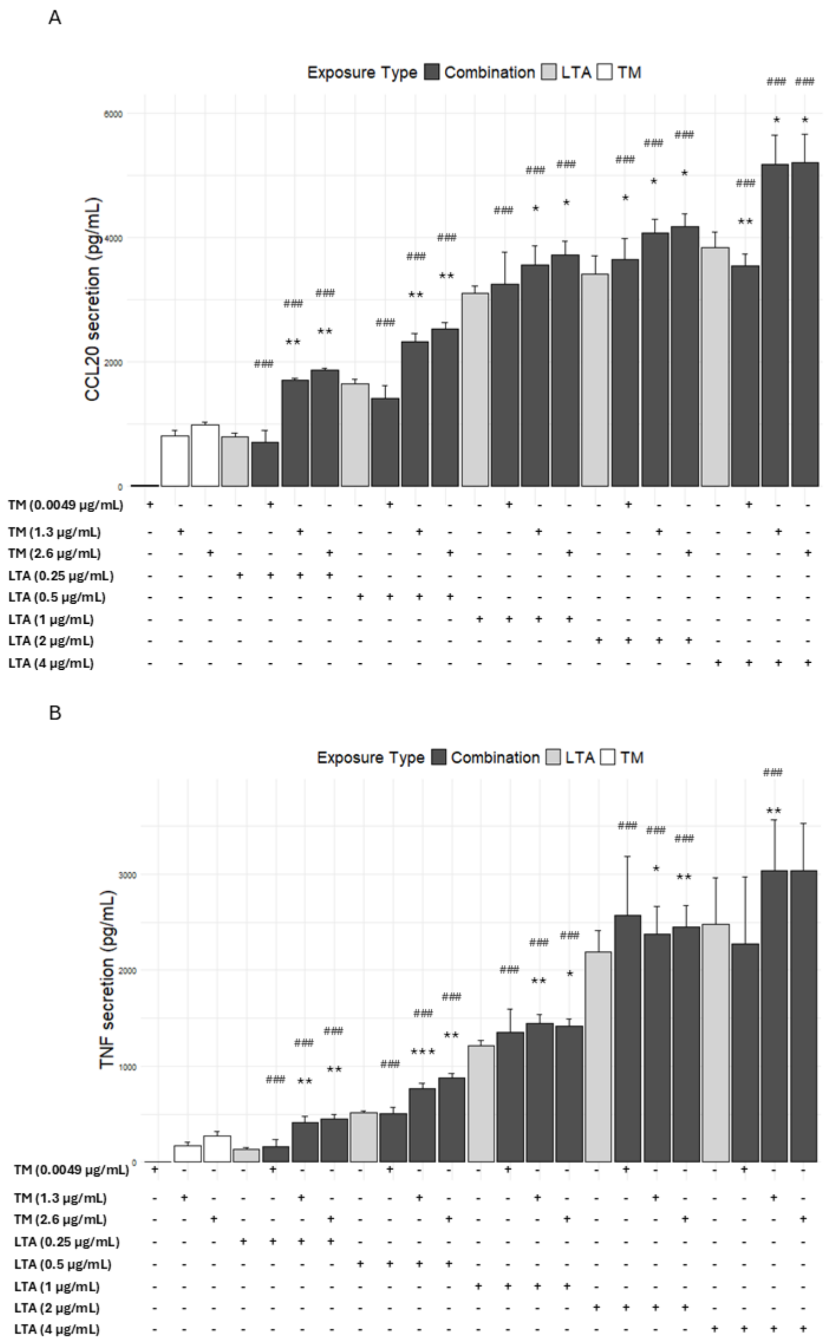

Figure S4 Release of inflammatory markers. THP-1 cells were exposed to three different concentrations of shrimp tropomyosin (TM) and five concentrations of lipoteichoic acid (LTA)

for 24 h and assessed for release of A) CCL20 and B) TNF. The bars represent the mean ± SEM, n=3. One-way ANOVA with Sidak's multiple comparison test was performed on log-transformed data. Significant differences between LTA and combined exposure are indicated by *, TM and combined exposure indicated by #, * or # $p < 0.05$, ** or ## $p < 0.005$ and *** or ### $p < 0.001$.

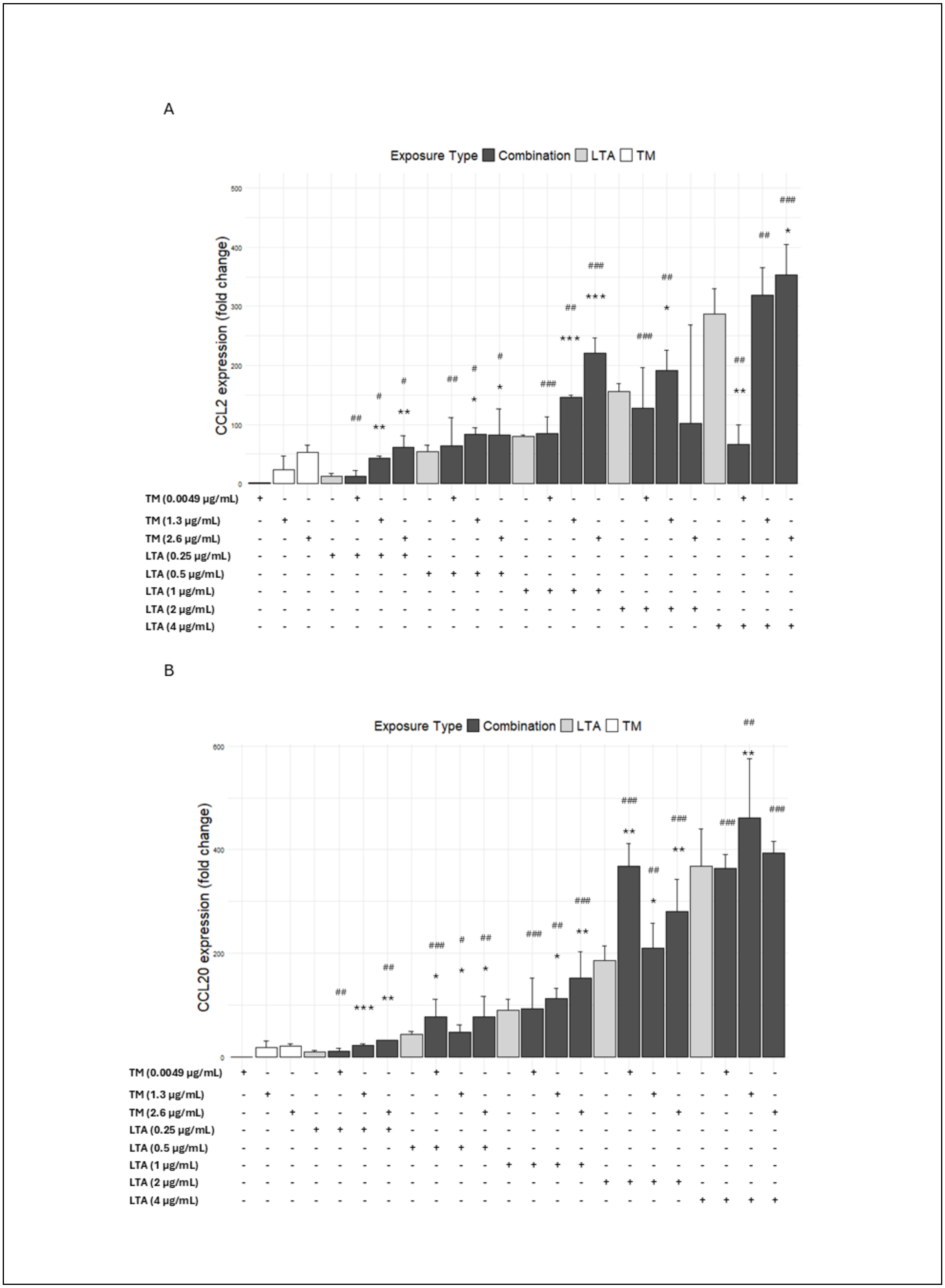

Figure S5 Expression of proinflammatory markers. THP-1 cells were exposed to three different concentrations of shrimp tropomyosin (TM) and five concentrations of lipoteichoic acid (LTA)

for 24 h and assessed for expression of A) *CCL2* and B) *CCL20*. The bars represent the mean ± SEM, n=3. One-way ANOVA with Sidak's multiple comparison test was performed on log-transformed data. Significant differences between LTA and combined exposure are indicated by *, TM and combined exposure indicated by #, * or # $p < 0.05$, ** or ## $p < 0.005$ and *** or ### $p < 0.001$.

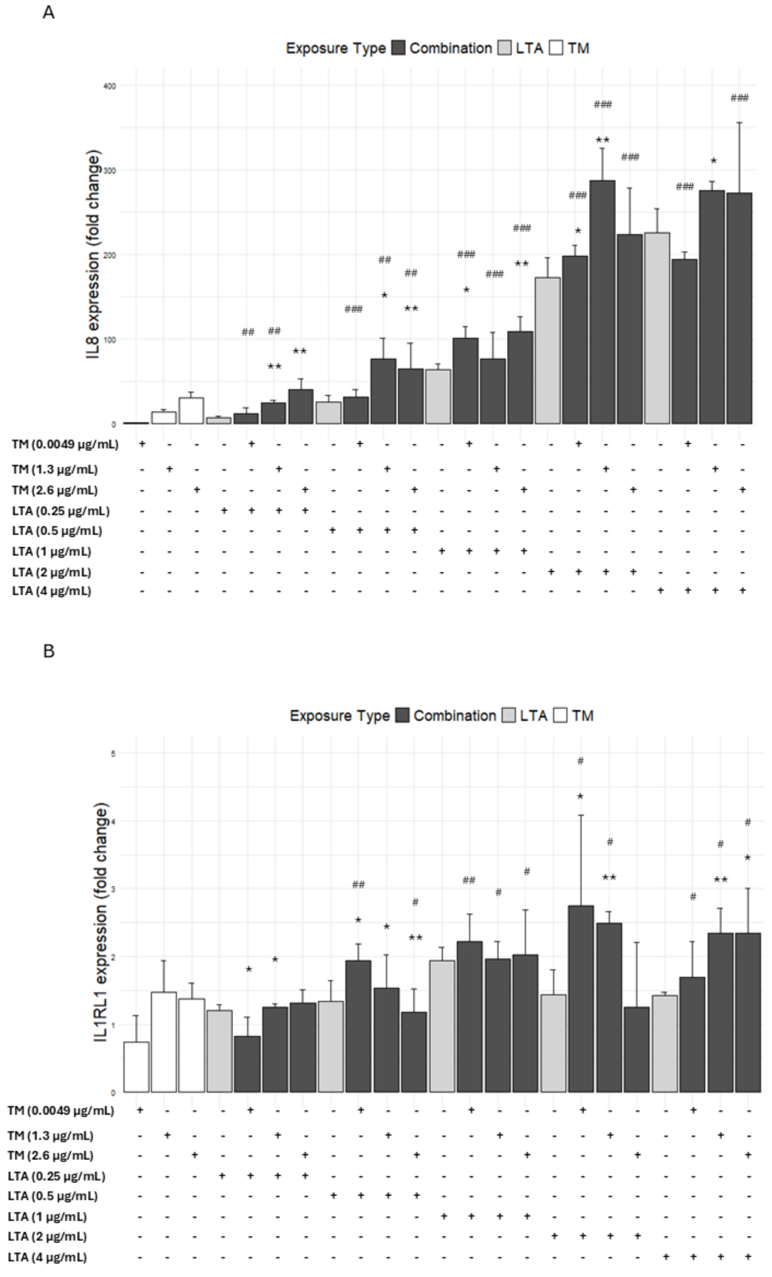

Figure S6 Expression of A) *IL8* and B) *IL1RL1* in THP-1 cells following 24 h of exposure to shrimp tropomyosin (TM) and lipoteichoic acid (LTA). The bars represent the mean ± SEM, n=3.

One-way ANOVA with Sidak’s multiple comparison test was performed on log-transformed data. Significant differences between LTA and combined exposure are indicated by *, TM and combined indicated by #, * or # $p < 0.05$, ** or ## $p < 0.005$ and *** or ### $p < 0.001$.

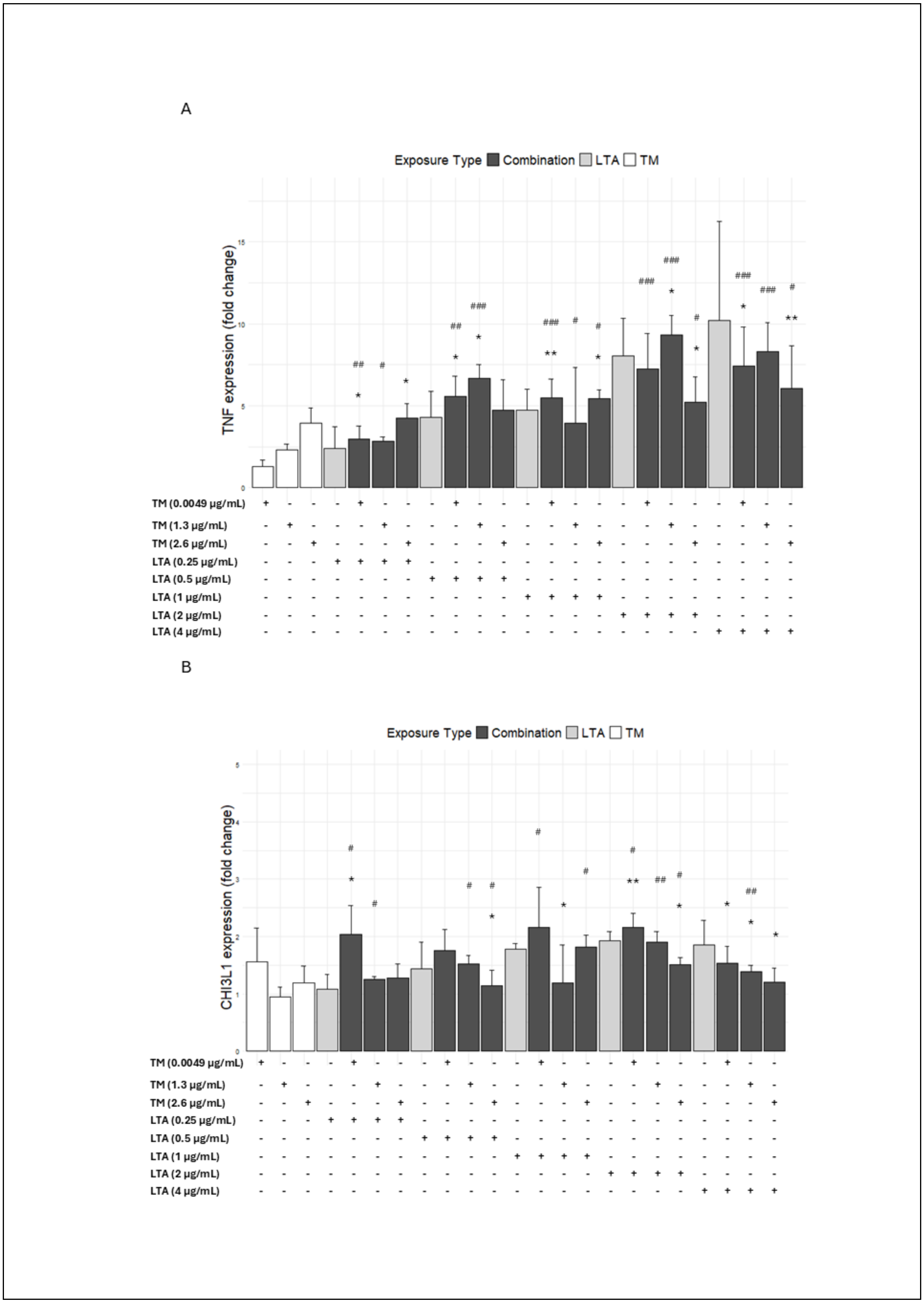

Figure S7 Expression of A) *TNF* and B) *CHI3L1 in* THP-1 cells following 24 h of exposure to shrimp tropomyosin (TM) and lipoteichoic acid (LTA). The bars represent the mean ± SEM, n=3.

One-way ANOVA with Sidak's multiple comparison test was performed on log-transformed data. Significant differences between LTA and combined exposure are indicated by *, TM and combined indicated by #, * or # $p < 0.05$, ** or ## $p < 0.005$ and *** or ### $p < 0.001$.

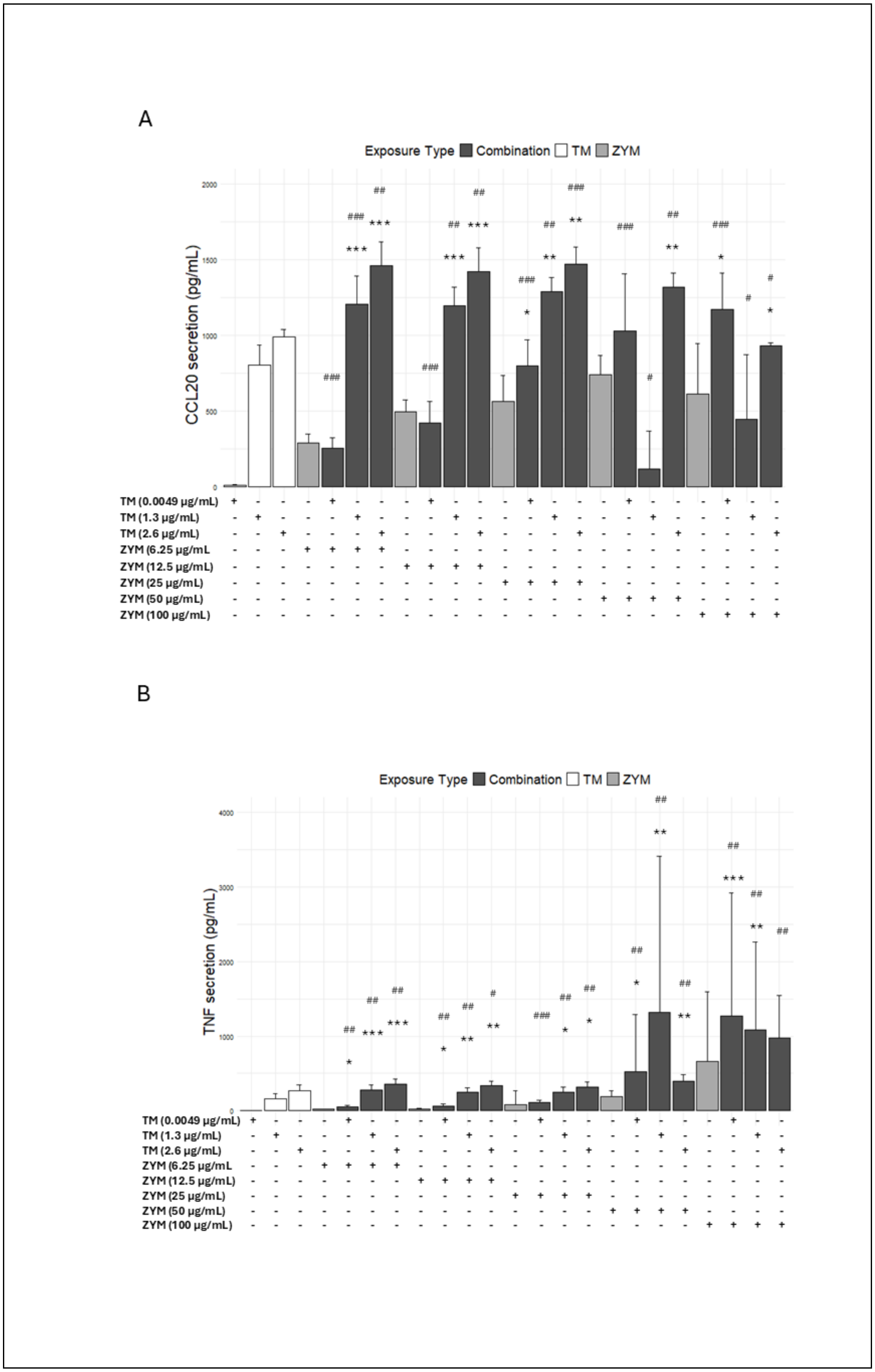

Figure S8 The release of proinflammatory markers in THP-1 cells following 24 h of exposure to shrimp tropomyosin (TM) and zymosan (ZYM). A) CCL20 and B) TNF. The bars represent the

mean ± SEM, n=3. One-way ANOVA with Sidak's multiple comparison test was performed on log-transformed data. Significant differences between ZYM and combined exposure are indicated by *, TM and combined exposure indicated by #, * or # $p < 0.05$, ** or ## $p < 0.005$ and *** or ### $p < 0.001$.

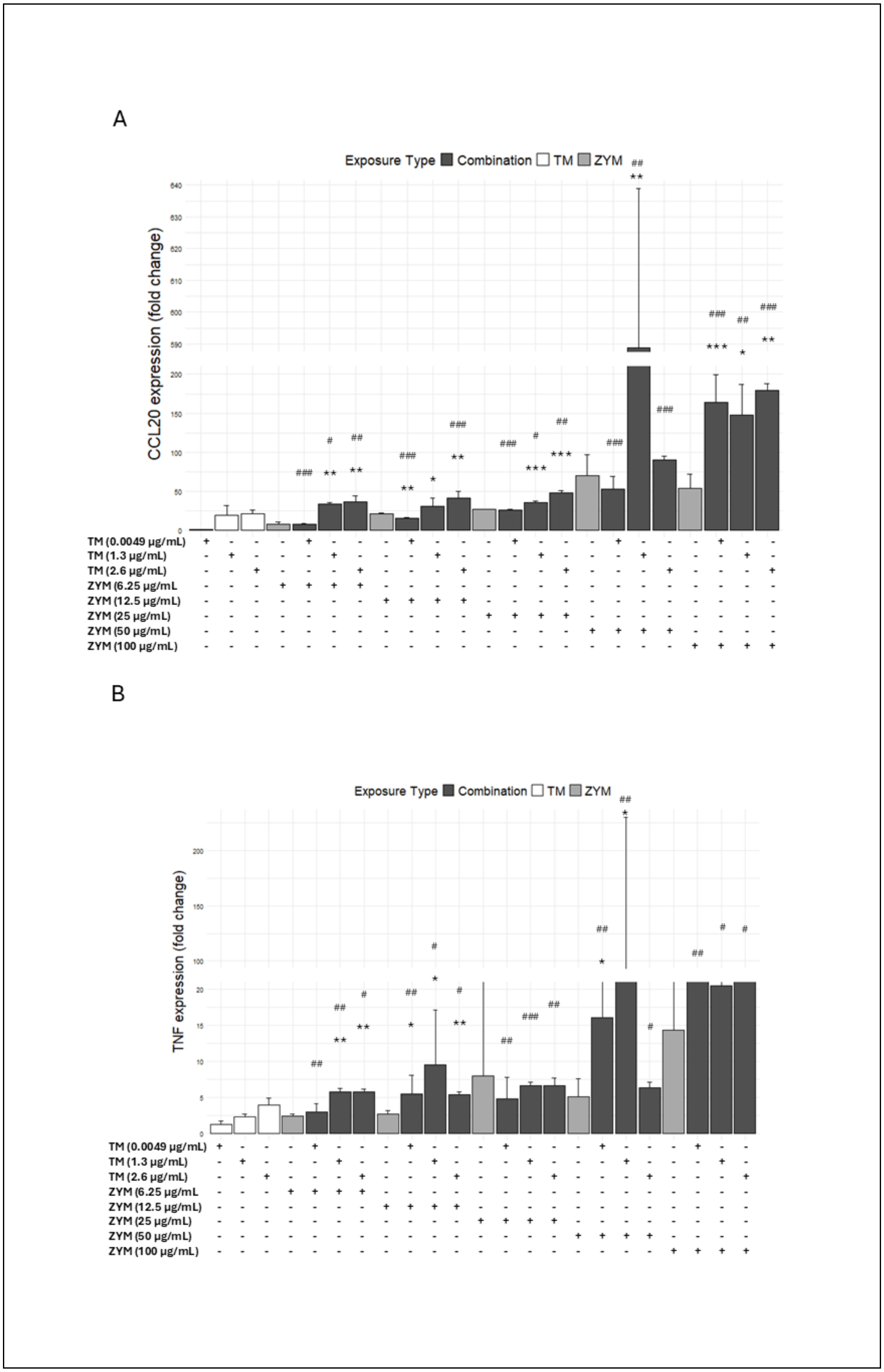

Figure S9 Expression of A) *CCL20* and B) *TNF* in THP-1 following 24 h of exposure to shrimp tropomyosin (TM) and Zymosan (ZYM). The bars represent the mean ± SEM, n=3. One-way

ANOVA with Sidak's multiple comparison test was performed on log-transformed data. Significant differences between ZYM and combined exposure are indicated by *, TM and combined indicated by #, * or # $p < 0.05$, ** or ## $p < 0.005$ and *** or ### $p < 0.001$.

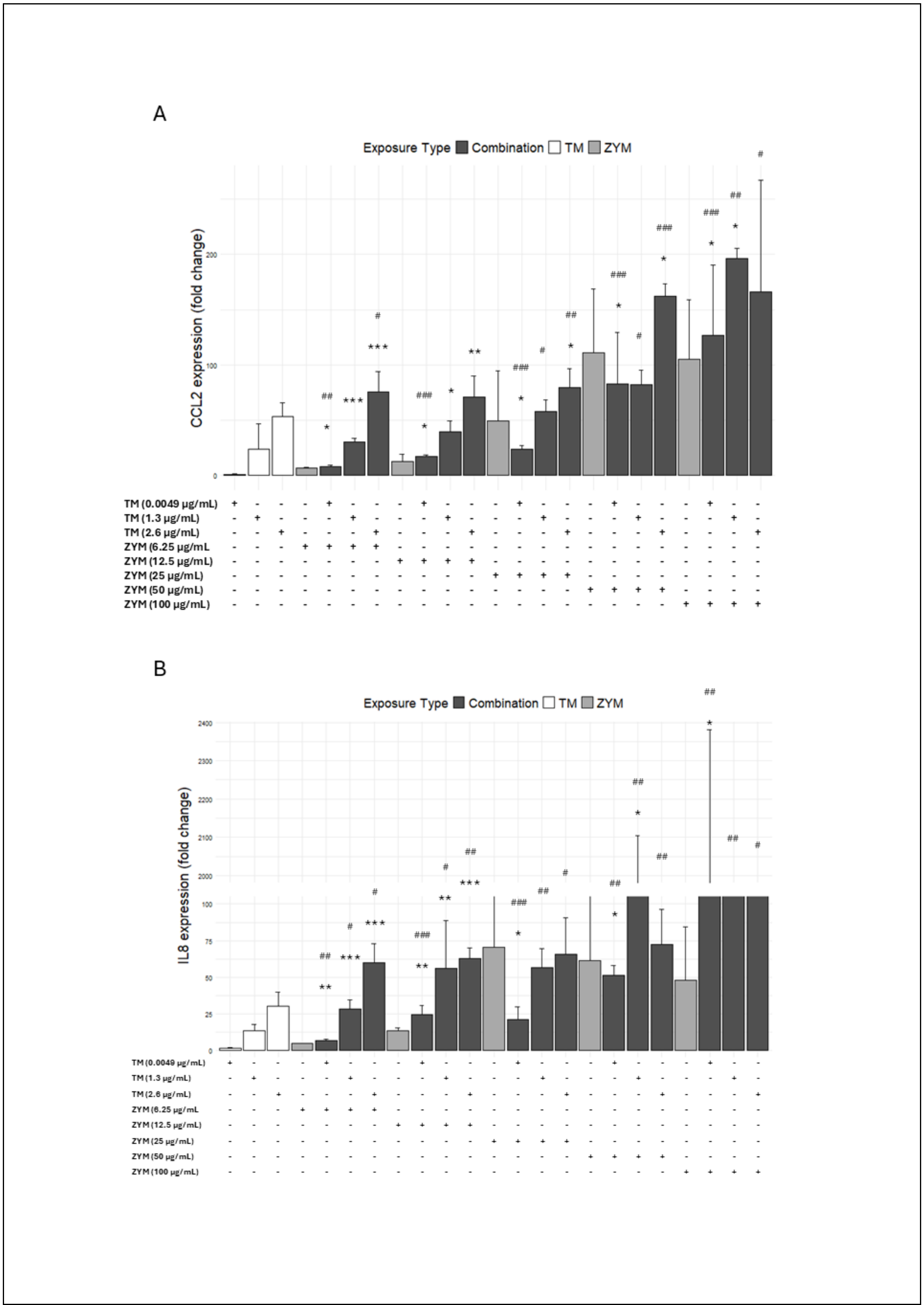

Figure S10 Expression of A) *CCL2* and B) *IL8* in THP-1 following 24 h of exposure to shrimp tropomyosin (TM) and Zymosan (ZYM). The bars represent the mean ± SEM, n=3. One-way

ANOVA with Sidak's multiple comparison test was performed on log-transformed data. Significant differences between ZYM and combined exposure are indicated by *, TM and combined indicated by #, * or # $p < 0.05$, ** or ## $p < 0.005$ and *** or ### $p < 0.001$.

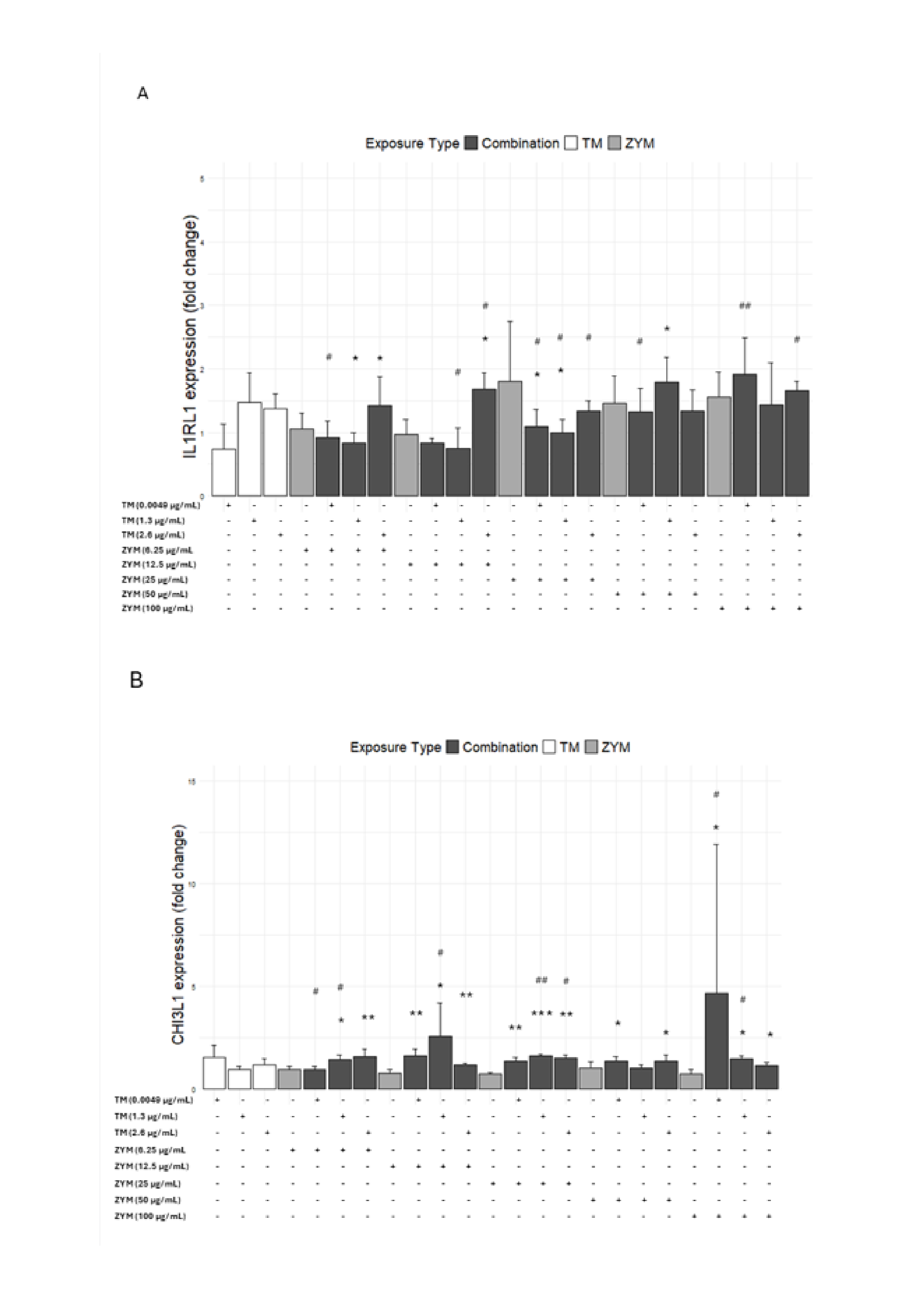

Figure S11 Gene expression of A) *IL1RL1* and B) *CHI3L1* in THP-1 cells after exposure to shrimp tropomyosin (TM) and zymosan (ZYM). The bars represent the mean ± SEM, n=3. One-

way ANOVA with Sidak's multiple comparison test was performed on log-transformed data. Significant differences between ZYM and combined exposure are indicated by *, TM and combined indicated by #, * or # $p < 0.05$, ** or ## $p < 0.005$ and *** or ### $p < 0.001$.

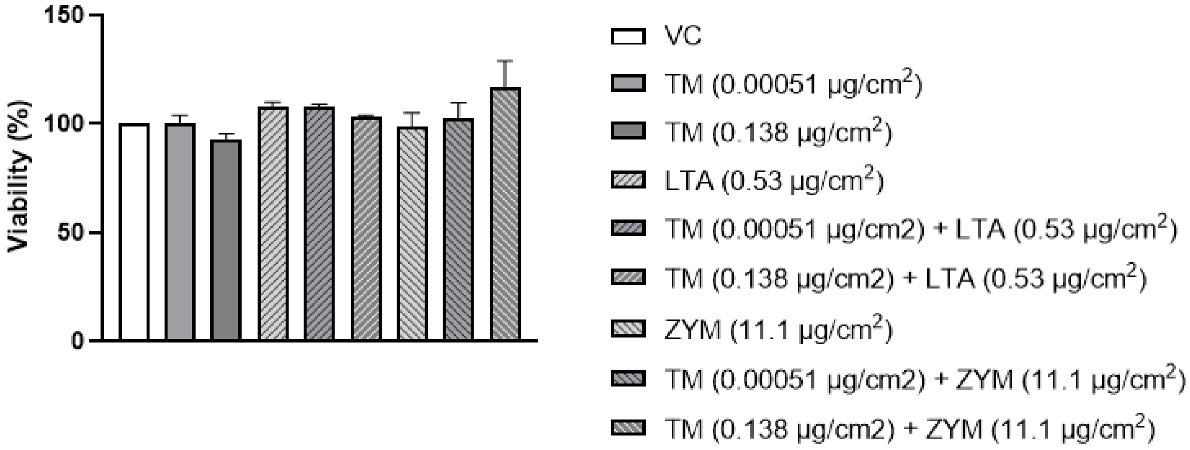

Figure S12. Viability of co-culture cells after individual and combined exposure to two different concentrations of shrimp tropomyosin (TM) and lipoteichoic acid (LTA) or zymosan (ZYM). VC indicates vehicle control.

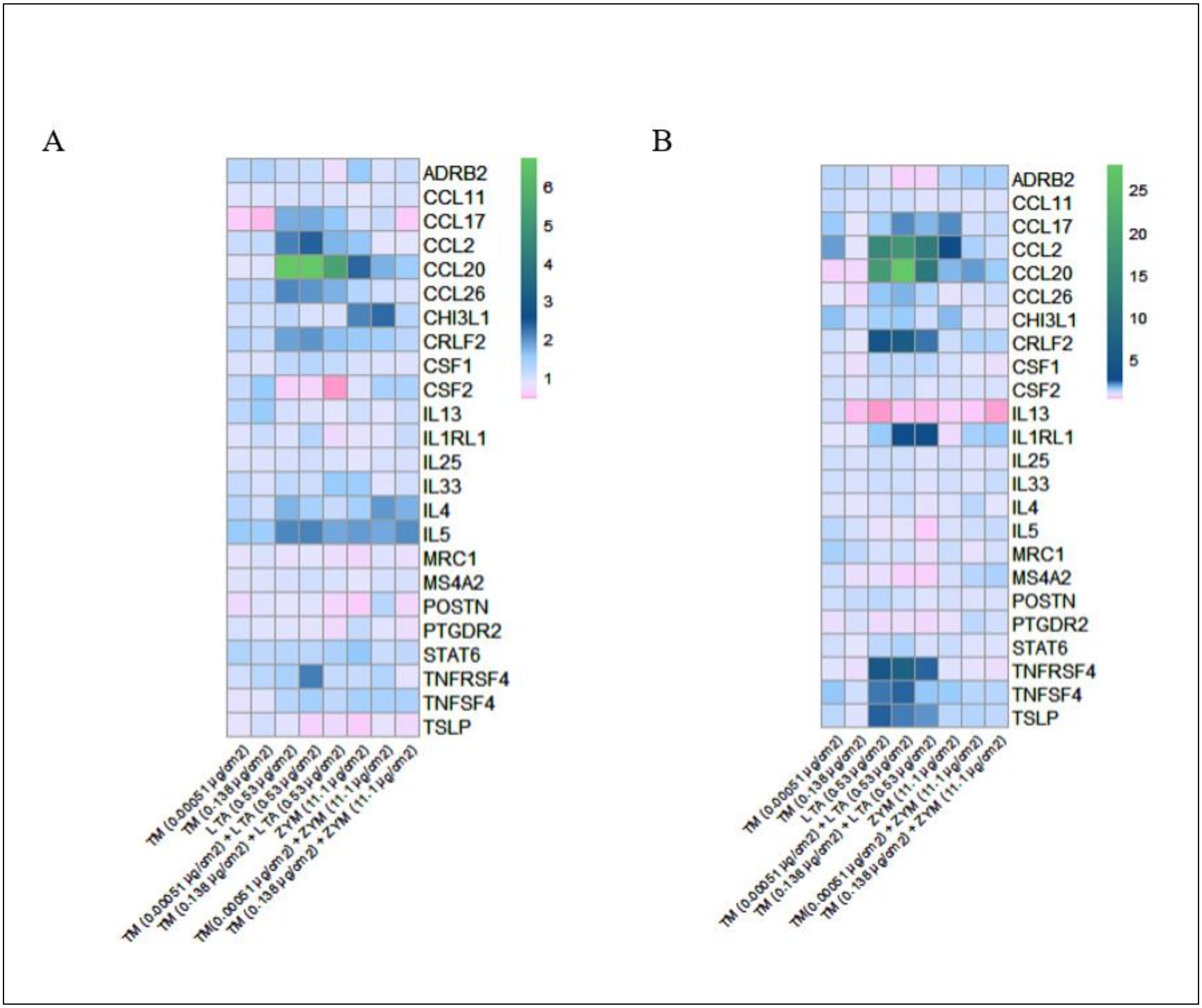

Figure S13 Heatmap showing the gene expression levels of asthma and allergy-related biomarker genes following a 24-hour combined exposure to shrimp tropomyosin (TM) and lipoteichoic acid (LTA) or zymosan (ZYM) in an alveolar co-culture model. The plotted values are the mean fold change of gene expression relative to the vehicle control. A) The apical compartment of the coculture comprised A549, differentiated THP-1 macrophages, and EA.hy926 cells. B) Basolateral compartment (dTHP-1) of the co-culture model.
