# Supplementary material for: Combined exposure to shrimp tropomyosin and microbial components triggers enhanced allergic and inflammatory responses in vitro
Source: Front Allergy. 2025 Nov 24;6:1654600. doi: 10.3389/falgy.2025.1654600 (PMC12683351; doi:10.3389/falgy.2025.1654600)
Supplement: Supplementary file 3 [file Datasheet3.pdf]

Supplementary data 1: Materials and methods

Table S1 Culture conditions *

Cell line	Medium	Fetal Bovine Serum (FBS) (%)	Supplement
EA.hy926	Dulbecco's Modified Eagle's Medium (DMEM)	10	25 mM HEPES
A549	DMEM	10	-
THP-1	Roswell Park Memorial Institute 1640 (RPMI-1640)	10	50 µM β-mercaptoethanol
Coculture 10 % of FBS	75 % DMEM<br>15 % RPMI-1640<br>10 % Iscove's Modified Dulbecco's Medium (IMDM)	10	25 mM HEPES
Coculture 1 % of FBS	75 % DMEM<br>15 % RPMI-1640<br>10 % IMDM	1	25 M HEPES

DMEM, RPMI 1640, IMDM, HEPES and β-mercaptoethanol (Gibco, ThermoFisher Scientific). FBS (Biosite Inc., San Diego, CA, USA). * Adopted and modified from (Chary et al., 2019).

Table S2 Tropomyosin dose calculation.

Scenario	TM conc. (µg/m^3)	Employment (yrs.)	Daily dose (µg/cm^2)	Cumulative dose (µg/cm^2)
Median	0.3	3.3	6.43×10^{-7}	5.09×10^{-4}
Maximum	7.07	38	1.52×10^{-5}	1.38×10^{-1}

Tropomyosin dose calculation.

Modified lung deposition formula from (Antonini et al., 2010).

$$\text{"Daily Exposure Dose "} \left(\mu\frac{\text{g}}{\text{cm\^{}2}}\right) = \text{TM Conc.}\left(\mu\frac{\text{g}}{\text{cm\^{}3}}\right) \times \text{Breathing volume(3 m\^{}3) / Lung surface area (140 m\^{}2)}$$

$$\text{"Exposure dose over emp. period"} \left(\mu\frac{\text{g}}{\text{cm\^{}2}}\right) = \text{Daily Exposure Dose } \left(\mu\frac{\text{g}}{\text{cm\^{}2}}\right) \times 5 \left(\frac{days}{week}\right) \times 48\frac{weeks}{year} \times emp.\,(yrs)$$

Daily exposure and exposure dose over the employment period based on the median tropomyosin exposure level.

$$\text{Daily Exposure Dose } \left(\mu\frac{\text{g}}{\text{cm\^{}2}}\right) = 0.3 \left(\mu\frac{\text{g}}{\text{cm\^{}3}}\right) \times \text{3 m\^{}3 / 140 m\^{}2}$$

Daily Exposure Dose = 0.000000643 μg/cm^2

$$\text{Exposure dose over emp. period} \left(\mu\frac{\text{g}}{\text{cm\^{}2}}\right) = 0.000000643 \left(\mu\frac{\text{g}}{\text{cm\^{}2}}\right) \times 5 \left(\frac{days}{week}\right) \times 48\frac{weeks}{year} \times 3.3\,(yrs)$$

Exposure dose over emp. period = 0.00051 μg/cm^2

Daily exposure and exposure dose over the employment period based on the maximum tropomyosin exposure level.

$$\text{Daily Exposure Dose " } \left(\mu\frac{\text{g}}{\text{cm\^{}2}}\right) = 7.07 \left(\mu\frac{\text{g}}{\text{cm\^{}3}}\right) \times \text{3 m\^{}3 / 140 m\^{}2}$$

Daily Exposure Dose = 0.00001515 μg/cm^2

$$\text{Exposure dose over emp. period} \left(\mu\frac{\text{g}}{\text{cm\^{}2}}\right) = 0.00001515 \left(\mu\frac{\text{g}}{\text{cm\^{}2}}\right) \times 5 \left(\frac{days}{week}\right) \times 48\frac{weeks}{year} \times 38\,(yrs)$$

Exposure dose over emp. period = 0.138 μg/cm^2

Table S3: Primer sequences used for the qPCR on RNA isolated from the THP-1 cells

Gene	Forward primer (5' → 3')	Reverse primer (5'→ 3')	RefSeq ID
CHI3L1	TGTACCCACATCATCTACAG	ACAGACAAGAGAGTCTTCAG	NM_001276.4
IL1RL1	ATTTAAGCAGAAACTACCCG	TTGCAATCCTTATACCACTG	NM_000877.4
CCL20	TATATTGTGCGTCTCCTCAG	GCTATGTCCAATTCCATTCC	NM_001130046.2
CCL2	AGACTAACCCAGAAACATCC	ATTGATTGCATCTGGCTG	NM_002982.4
IL8	GTTTTTGAAGAGGGCTGAG	TTTGCTTGAAGTTTCACTGG	NM_000584.4
TNF	CTCAGCCTCTTCTCCTTC	AGAAGATGATCTGCTGCC	NM_000594.4
B2M	AAGGACTGGTCTTTCTATCTC	GATCCCACTTAACTATCTTGG	NM_004048.4
HPRT1	ATAAFCCAGACTTTGTTGG	ATAGGACTCCAGATGTTTCC	NM_000194.3

Table S4 Genes included in custom asthma and allergy RT2 Profiler PCR Array

Gene code	Gene Name	RefSeq ID
ADRB2	Adrenoceptor beta 2	NM_000024.6
CCL11	C-C motif chemokine ligand 11	NM_002986.3
CCL17	C-C motif chemokine ligand 17	NM_002987.3
CCL2	C-C motif chemokine ligand 2	NM_002982.4
CCL20	C-C motif chemokine ligand 20	NM_004591.3
CCL26	C-C motif chemokine ligand 26	NM_001371936.1
CHI3L1	Chitinase-3-like protein 1	NM_001276.4
CRLF2	Cytokine receptor-like factor 2	NM_001012288.3
CSF2	Granulocyte-macrophage colony-stimulating factor	NM_000758.4
IL13	Interleukin13	NM_001354993.2
IL1RL1	Interleukin 1 receptor-like 1	NM_001282408.2
IL25	Interleukin 25	NM_172314.2
IL33	Interleukin 33	NM_001199641.2
IL4	Interleukin 4	NM_001354990.2
IL5	Interleukin 5	NM_000879.3
MRC1	Mannose receptor c-type 1	NM_002438.4
MS4A2	Membrane spanning 4-domains A2	NM_001256916.2
POSTN	Osteoblast specific factor	NM_001424173.1
PTGDR2	Prostaglandin D2 receptor 2	NM_004778.3
STAT6	Signal transducer and activator of transcription 6	NM_001178081.2
TNFRSF4	Tumor necrosis factor receptor superfamily member 4	NM_001410709.1
TNFSF4	Tumor necrosis factor ligand superfamily member 4	NM_001297562.2
TSLP	Thymic stromal lymphopoietin	NM_138551.5
B2M	Beta-2-microglobulin	NM_004048.4
HPRT1	Hypoxanthine phosphoribosyl transferase 1	NM_000194.3
TBP	TATA-box binding protein	NM_001172085.2
HMBS	Hydroxymethylbilane synthase	NM_001425063.1
TFRC	Transferrin receptor	NM_001313965.2
